# Phenotypic Disease Network Analysis to Identify Comorbidity Patterns in Hospitalized Patients with Ischemic Heart Disease Using Large-Scale Administrative Data

**DOI:** 10.3390/healthcare10010080

**Published:** 2022-01-01

**Authors:** Dejia Zhou, Liya Wang, Shuhan Ding, Minghui Shen, Hang Qiu

**Affiliations:** 1 Big Data Research Center, University of Electronic Science and Technology of China, Chengdu 611731, China; zhoudj@std.uestc.edu.cn (D.Z.); hbigdata@uestc.edu.cn (L.W.); 2 School of Computer Science and Engineering, University of Electronic Science and Technology of China, Chengdu 611731, China; 3 School of Electrical and Computer Engineering, Cornell University, Ithaca, NY 14853, USA; sd925@cornell.edu; 4 Health Information Center of Sichuan Province, Chengdu 610041, China; Shenmh@schnic.cn

**Keywords:** ischemic heart disease, comorbidity, phenotypic disease network, network analysis, physical comorbidity, mental comorbidity

## Abstract

Ischemic heart disease (IHD) exhibits elevated comorbidity. However, few studies have systematically analyzed the comorbid status of IHD patients with respect to the entire spectrum of chronic diseases. This study applied network analysis to provide a complete picture of physical and mental comorbidities in hospitalized patients with IHD using large-scale administrative data. Hospital discharge records from a provincial healthcare database of IHD inpatients (*n* = 1,035,338) and one-to-one matched controls were included in this retrospective analysis. We constructed the phenotypic disease networks in IHD and control patients and further assessed differences in comorbidity patterns. The community detection method was applied to cluster diagnoses within the comorbidity network. Age- and sex-specific patterns of IHD comorbidities were also analyzed. IHD inpatients showed 50% larger comorbid burden when compared to controls. The IHD comorbidity network consisted of 1941 significant associations between 71 chronic conditions. Notably, the more densely connected comorbidities in IHD patients were not within the highly prevalent ones but the rarely prevalent ones. Two highly interlinked communities were detected in the IHD comorbidity network, where one included hypertension with heart and multi-organ failures, and another included cerebrovascular diseases, cerebrovascular risk factors and anxiety. Males exhibited higher comorbid burden than females, and thus more complex comorbidity relationships were found in males. Sex-specific disease pairs were detected, e.g., 106 and 30 disease pairs separately dominated in males and females. Aging accounts for the majority of comorbid burden, and the complexity of the comorbidity network increased with age. The network-based approach improves our understanding of IHD-related comorbidities and enhances the integrated management of patients with IHD.

## 1. Introduction

Ischemic heart disease (IHD) has become the leading cause of morbidity and mortality globally, representing 16.2% of total deaths and 7.2% of all disability-adjusted life years (DALYs) in 2019 [1]. In China, the number of patients experiencing IHD reached 11 million in 2017 [2], and deaths from IHD increased from 745,000 in 1990 to 1,394,000 in 2013 [3].

IHD co-occurs with other medical conditions far more commonly than expected by chance. Previous studies have reported high comorbidity between IHD and other chronic physical and mental disorders, such as hypertension [4,5,6,7], diabetes [7,8,9], chronic obstructive pulmonary disease (COPD) [7,10], and depression [11,12]. In fact, a study of 92,186 IHD patients in the United Kingdom observed that 84% of patients with IHD had one or more comorbidities, of which 47.1% had hypertension and 15.7% had diabetes [7]. Another meta-analysis based on the Charlson Comorbidity Index (CCI) found that diabetes and a history of prior myocardial infarction were the two most common comorbidities in patients with IHD [9]. Comorbid conditions in IHD have been shown to be correlated with slower recovery, lower quality of life, higher healthcare demands, and increased mortality [10,13,14]. Two separate studies [15,16] found a relationship between an incremental rise in CCI score and mortality in patients with stable IHD, suggesting that incremental increases in CCI score were correlated with worse outcomes. Therefore, understanding the patterns of diseases that co-exist with IHD is important for disease management and improving the quality of healthcare.

Despite the growing amount of evidence that IHD has high co-existing morbidity burden, previous studies investigating IHD comorbidity have been predominately focused on a few common diseases and interpreted them using prevalence statistics and correlation analysis [7,9,10,12,13], without a comprehensive investigation of associations between comorbidities in patients with IHD, and most of these studies were conducted in Western countries [15,16,17,18]. There is still a significant gap in knowledge regarding the comorbidity patterns of IHD, especially among Chinese individuals, who tend to exhibit different lifestyles and eating habits from those observed in Western countries. Moreover, the differences in comorbid patterns between IHD and non-IHD patients have rarely been reported.

Recent developments in network medicine have led to a proliferation of studies that used complex networks for medical research to explore the comorbidity of various diseases [19,20,21,22,23,24,25,26,27,28]. For instance, Hidalgo et al. [29] used network methods to study the disease progression. A case–control cohort study applied network-based analysis to explore the associations between multiple comorbidities of COPD [30]. Further, Mu et al. [31] investigated the comorbidity patterns of hepatocellular carcinoma based on a comorbidity network. 

The increase in these types of studies is largely attributable to a massive increase in administrative data, which provides the opportunity to simultaneously evaluate the entire spectrum of diagnoses for comorbidity in a single study. The diagnosis codes contained in administrative datasets have great potential in helping to understand the nature of comorbidities. However, to our knowledge, no study has applied network theories and administrative data to systematically investigate the physical and mental comorbidities of IHD, which may allow us to discover previously unrecognized relationships among diseases.

Therefore, in this study, we aimed to systematically analyze the comorbid status of hospitalized patients with IHD along the entire spectrum of chronic diseases using a network approach based on large-scale administrative health data and compared with control individuals.

## 2. Materials and Methods

### 2.1. Study Design, Data Source and Study Population

This was a large-sample, retrospective study based on anonymized hospital discharge records (HDRs) collected from all of the secondary and tertiary hospitals in Sichuan Province, accessed from a provincial healthcare database. All of the above-mentioned hospitals must upload HDRs according to a uniform format and quality requirements, through the Network Direct Reporting System of the Health Information Center of Sichuan Province [32]. Each HDR consists of a unique patient identifier, sex, age, date of admission and discharge, hospitalization expenses, primary discharge diagnosis and up to 15 secondary diagnoses. All diagnoses are specified by the ICD-10 (International Classification of Diseases, 10th Revision). Since 2015, the system has been undergoing preliminary cleaning, recoding, and verification to improve data integrity, accuracy of disease coding, and reduce heterogeneity [33].

In total, 47.36 million hospitalizations during the entire study period (1 January 2015–31 December 2019) were reported, which offered reliable, systematic, and complete data for comorbidity detection. In the present study, IHD cases were defined as inpatients living in Sichuan Province, aged ≥35 years, with a diagnosis of I20–I25, and no death reported during the entire study period, for a total of 1,035,338 unique IHD cases. For each IHD case, a one-to-one matched control was randomly selected from inpatients without IHD during the same study period. Controls were individually matched to the index patient by year of birth (±2 years), sex, discharge date, and level of hospital [34,35,36].

This study was approved by the Ethics Committee of Health Information Center of Sichuan Province. The requirement to obtain informed consent was waived because of the secondary nature of the de-identified data in the retrospective study design.

### 2.2. Identification of Comorbidities and Comorbid Burden

We examined all diagnoses using the ICD-10 codes at three digits and applied the chronic condition indicator to differentiate between acute and chronic diseases [37,38]. Diagnostic codes from chapters XIX (Injury, poisoning, and certain other consequences of external causes, S00–T88), XX (External Causes of Morbidity, V00–Y99), and XXI (Factors influencing health status and contact with health services, Z00–Z99) were excluded, since they are not diseases or are general symptoms. To obtain more reliable estimates, rarely diagnosed diseases (prevalence < 1%) were also excluded [24,25,39,40]. Using these criteria, 71 and 63 chronic diseases with a prevalence of ≥1% were identified as comorbidities in IHD inpatients and controls, respectively, where 57 comorbidities were common to both cases and controls (Supplementary Table S1). We defined comorbid burden as the number of comorbidities that each patient was diagnosed with during the study period.

### 2.3. Estimation of Enrichment Comorbidities in IHD

The odds ratio (OR) of each comorbidity, as well as the corresponding 95% confidence intervals (95% CIs), were calculated to measure the strength of chronic disease co-existence with IHD inpatients when compared to controls. A Bonferroni correction was applied to control for repeated testing with a *p* value for statistical significance of an OR being 0.05/71. Enrichment comorbidities in IHD were defined as diseases which were more likely to occur in IHD inpatients (ORs > 1 with *p* values < 0.05/71), and their prevalence increased over 0.5 times when compared to controls.

### 2.4. Comorbidity Network Construction and Community Detection

We constructed the phenotypic disease networks (PDNs) in IHD cases and controls, with each node representing a disease diagnosis (comorbidity) and each edge denoting the co-occurrence relationship between pair-wise comorbidities [29]. 

We used the observed-to-expected ratio (OER) to measure comorbidity strength [29,41,42,43]. *OER_ij_*, which is the comorbidity strength of disease pair *i* and *j*, is the ratio of observed prevalence of disease pair *i* and *j* (O) to the expected prevalence (E) based on the product of disease *i* prevalence and disease *j* prevalence:

(1) $$OER_{\mathrm{ij}}=\frac{C_{ij}N}{C_{i}C_{j}}$$

where *C_ij_* is the number of patients exhibiting both diseases, *N* is the total number of patients in the population, and *C_i_* and *C_j_* are the number of patients diagnosed with diseases *i* and *j*, respectively. Generally, an OER >1 means that the pair-wise diseases co-occurred more often in the same patients than excepted by chance, while an OER <1 indicates the pair-wise diseases tend to be mutually exclusive. As we were interested in comorbid relationships, disease pairs with OERs >1 (according to the corresponding 99% CI to calculate statistical significance) were included in the network. A discussion on the confidence interval and statistical significance of the OER can be found in the Supplementary Material.

To measure the structural properties of the PDNs, five network metrics, including diameter, network density, degree centrality, closeness centrality, and average nearest neighbor degree, were adopted. Diameter is the maximum weighted distance between any two nodes in the disease network. The density of a network is the proportion of edges in the network, and it measures how compact the network is, with a higher network density indicating more associations between diseases. The degree centrality of a disease (node) denotes the number of direct connections with other diseases. The closeness centrality of a disease is the reciprocal of the sum of the length of the shortest paths between a disease and all other diseases in the graph. A disease with higher closeness has a higher risk of being diagnosed with other diseases in fewer steps. The average nearest neighbor degree of a disease measures dependencies between degrees of neighbor nodes in a network.

To identify the subsets of potential comorbidities that were more closely related, community detection was conducted based on the fast unfolding cluster algorithm, which modeled the highest modularity score among different clustering layers [44]. 

### 2.5. Age- and Sex-Specific Comorbidity Patterns in IHD

To investigate sex (male vs. female) and age (35–59, 60–69, 70–79 and ≥80 years) differences in comorbidity patterns in IHD, we constructed the comorbidity network in each subgroup. A pair-wise *t* test and one-way analysis of variance (ANOVA) were applied to analyze the sex and age differences in network properties, respectively.

All analyses and visualizations were conducted using Python 3.7 and Cytoscape 3.8.2.

## 3. Results

### 3.1. Comorbidity Status

**Figure 1:**
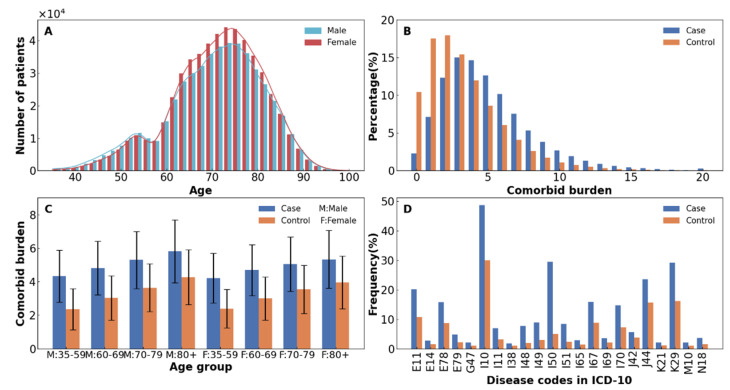
Demographic characteristics and comorbidity statistics. (**A**) Sex-specific age distribution for IHD patients. (**B**) Distribution of the number of comorbid burdens in patients with IHD and controls. (**C**) Mean number of comorbidities in IHD patients and controls by sex and age. The error bar represents mean ± standard deviation. (**D**) Enrichment comorbidities in IHD patients: prevalence of diagnosis ≥1% in both case and control, prevalence increased over 0.5 times increase in case (color blue) than controls (color orange). E11: diabetes mellitus; E14: unspecified diabetes mellitus; E78: disorders of lipoprotein metabolism and other lipidemias; E79: disorders of purine and pyrimidine metabolism; G47: sleep disorders; I10: essential (primary) hypertension; I11: hypertensive heart disease; I38: endocarditis, valve unspecified; I48: atrial fibrillation and flutter; I49: other cardiac arrhythmias; I50: heart failure; I51: complications and ill-defined descriptions of heart disease; I65: occlusion and stenosis of precerebral arteries, not resulting in cerebral infarction; I67: other cerebrovascular diseases; I69: sequelae of cerebrovascular disease; I70: atherosclerosis; J42: unspecified chronic bronchitis; J44: other chronic obstructive pulmonary disease; K21: gastro-esophageal reflux disease; K29: gastritis and duodenitis; M10: gout; N18: chronic renal failure.

A total of 1,035,338 IHD inpatients survived throughout the study period. The sample had a mean age of 71 years at enrollment and 51.63% were female (Figure 1A and Supplementary Figure S1). IHD patients had a higher comorbid burden than controls, e.g., the mean number of comorbidities (5.00 vs. 3.35) and the proportion of patients with at least two comorbidities (90.59% vs. 72.02%) were larger in cases when compared to controls (Figure 1B). Males demonstrated larger numbers of comorbidities than females, especially in individuals over 70 years old (Figure 1C). Among the 71 comorbidities with a prevalence of ≥1% in cases, 63 chronic diseases were statistically more likely to co-occur with IHD patients than the controls (ORs >1 with *p*-values < the Bonferroni corrected threshold), as shown in Supplementary Table S1. The highest OR was for heart disease, including heart failure (I50, OR = 8.02, 95% CI: 7.94–8.10), atrial fibrillation and flutter (I48, OR = 4.33, 95% CI: 4.26–4.40), and complications and ill-defined descriptions of heart disease (I51, OR = 3.82, 95% CI: 3.77–3.88). Altogether, 22 diseases were enriched in IHD patients, showing a 0.5-fold increase in prevalence compared to controls (Figure 1D).

Table 1 presents the top 20 prevalent comorbidities, e.g., nearly half of IHD cases were comorbid with hypertension (I10, 48.64%) and three in ten had heart failure (I50, 29.39%) or gastritis and duodenitis (K29, 29.10%). Meanwhile, comorbidities with relatively lower prevalence but a higher likelihood of co-occurring in IHD patients compared with controls still existed. For example, other anxiety disorders (F41) had relatively lower prevalence in IHD patients (prevalence = 1.62%, 95% CI: 1.59–1.64%) but was more likely to co-occur with IHD (OR = 1.96, 95% CI: 1.91–2.01). A scatter plot depicting ORs for comorbid conditions in case over their prevalence estimates is shown in Supplementary Figure S2.

### 3.2. Comorbidity Network

**Figure 2:**
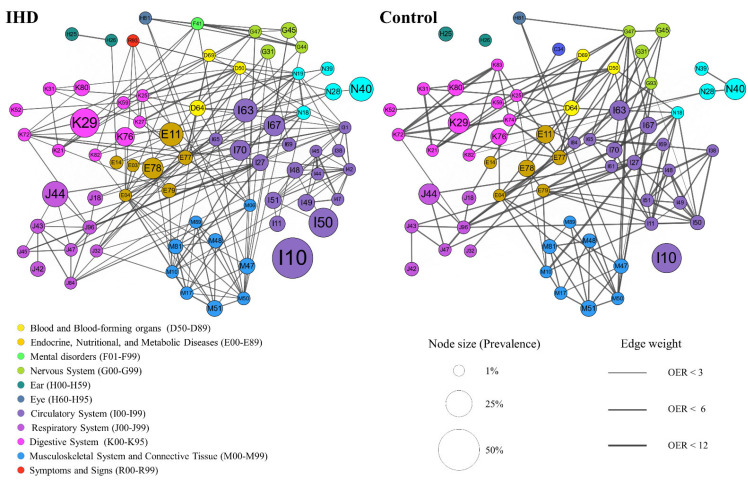
The strongest comorbidity associations in patients with IHD (**left** panel) and controls (**right** panel). Nodes represent comorbidities and are colored with disease chapters, node size indicates prevalence, and edge thickness represents observed-to-expected ratio (OER). Here, all statistically significant links where OERs > P90 are shown.

Overall, the comorbidity network in IHD patients was more complex than that in the controls, e.g., there were 71 nodes and 1941 statistically significant edges in the IHD comorbidity network, while there were only 63 nodes and 1372 edges in the controls. Atrioventricular and left bundle-branch block (I44), cardiac arrhythmias (I49), and paroxysmal tachycardia (I47) showed high centrality in the comorbidity network of IHD. The top three significant connections for the IHD group were atrioventricular and left bundle-branch block (I44) with other conduction disorders (I45), then anxiety disorders (F41) with sleep disorders (G47), and iron deficiency anemia (D50) with gastric ulcer (K25). However, in the control group, the top three most significant connections were other bacterial diseases (K72) with other diseases of biliary tract (K83), other disorders of brain (G93) with respiratory failure (J96), and gout (M10) with chronic kidney failure (N18). Hypertension (I10), the most prevalent comorbidity in IHD patients and the controls, was significantly comorbid with various diseases, such as diabetes mellitus (E11, E14), heart diseases (I44, I49, I50, I51), cerebrovascular diseases (I63, I65, I67, I69), arthropathies (M10, M17), dorsopathies (M47, M48, M50, M51), and kidney failure (N18, N19). For convenience, we visualized the comorbidity network by focusing on the strongest associations, as shown in Figure 2, which only includes the edges with OERs greater than the 90th percentile in IHD patients and controls, respectively (thresholds of 2.19 and 2.87, respectively). All the significant connections in IHD patients (ranked in descending order of OER value) are listed in Supplementary Table S2, where a clinician may search for conditions of interest and look up obvious and non-obvious comorbidity associations. 

**Figure 3:**
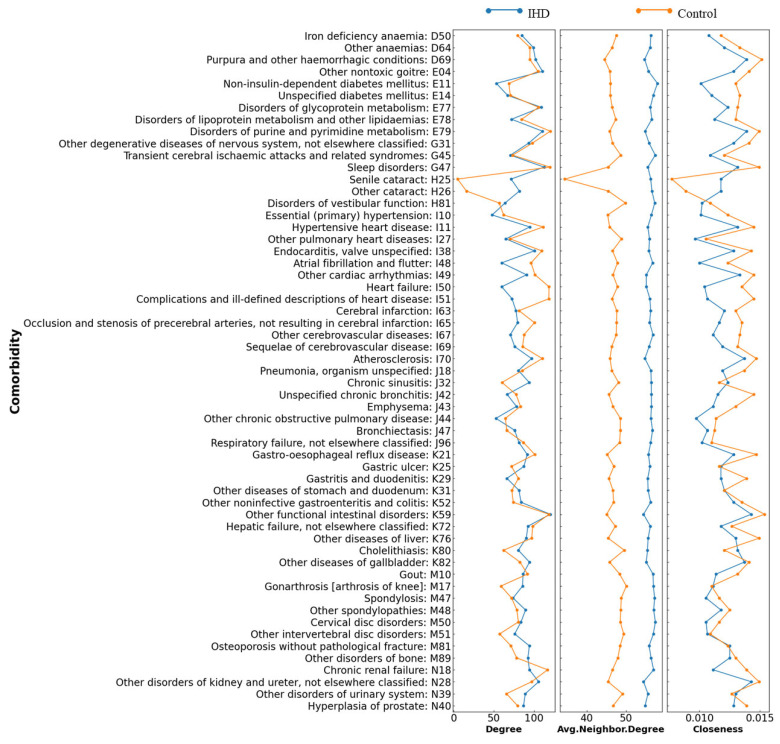
Three centrality measures visualized for IHD patients and control.

The nodes in the IHD network were more closely grouped and the relationships between diseases were more concentrated, as indicated by the elevated degree per node and the larger number of average degree per neighbor in the IHD network (Table 2). Most of nodes and their neighbors had larger connections in the comorbidity network of IHD patients than in the controls (Figure 3). For average neighborhood degree, the cases showed a significantly larger value (9.28 on average) than the controls, and the most important differences were observed in senile cataract (H25), diabetes mellitus (E11), and hypertension (I10). For closeness, the distribution was similar to degree, but due to the smaller diameter, the closeness of IHD cases was smaller than the controls.

**Figure 4:**
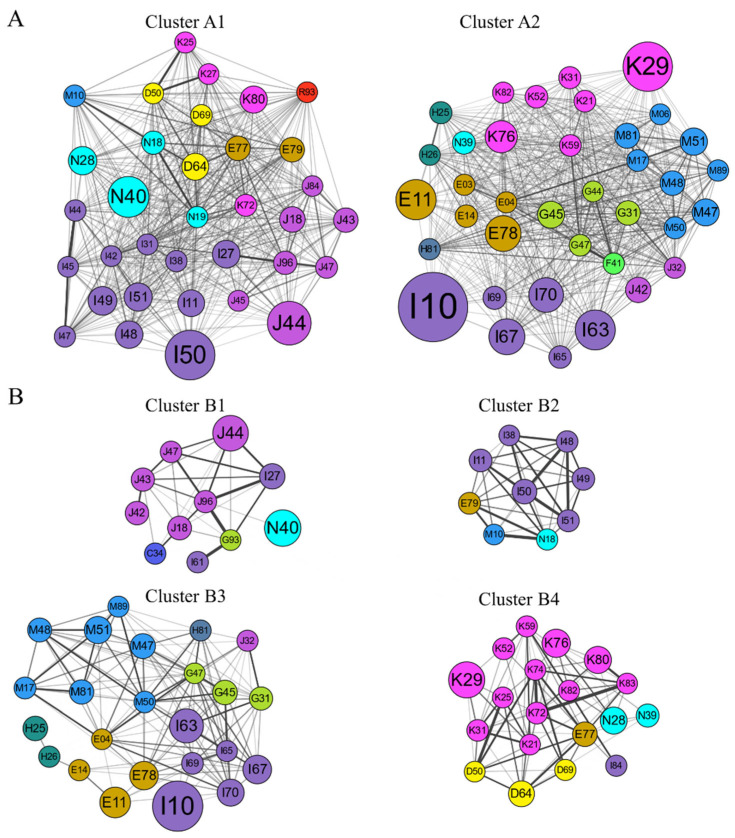
Clustering of comorbidity networks in patients with IHD (**A**) and controls (**B**). Nodes represent comorbidities and colored with disease chapters, node size indicates prevalence, and edge thickness represents observed-to-expected ratio (OER).

The comorbidity networks for IHD patients and controls contained distinct clusters (also referred to modules) of highly interlinked nodes, and the visual representation revealed a central theme (Supplementary Table S3). For IHD patients, two clusters were obtained (Cluster A1 and A2; see Figure 4), while for controls, four clusters were obtained (Clusters B1, B2, B3, and B4 in Figure 4). Cluster A1 was composed of 34 nodes connected by 525 edges around the theme of hypertension with heart, COPD, genitourinary diseases, and multi-organ failures. Note that in Cluster B1 (controls), respiratory disease, and in Cluster B2, hypertension, cluster together with heart disease; however, the prevalence and number of links were smaller. Cluster A2 was composed of 37 nodes and 613 edges in which comorbidities clustered around hypertensive cerebrovascular, endocrine, and digestive diseases, as well as other anxiety disorders (F41). For Cluster B3, comorbidities clustered around hypertensive cerebrovascular, endocrine disease, and digestive diseases, which was a pattern also observed in Cluster B4. 

### 3.3. Sex- and Age-Specific Comorbidity

Sex- and age-specific comorbidity networks in IHD patients are shown in Supplementary Figures S4 and S5, and the network metrics are shown in Table 3. The network of males was more complex than females, e.g., larger network diameter, mean degree, and network density was observed in the male network. The connection with the largest likely co-occurrence in the network of males was deficiency anemia (D50) with gastric ulcer (K25); OER = 8.09, 99% CI: 8.06–8.11), while the largest one in females was purpura and other hemorrhagic conditions (D69) with agranulocytosis (D70); OER = 11.26, 99% CI: 11.23–11.29). Meanwhile, sex differences in the strength of observed comorbidities also existed. Among the 61 comorbidities common to both sexes, 106 and 30 disease pairs involving different diseases systems were separately dominated in males (OER in males/OER in females ≥1.2) and females (OER in females/OER in males ≥1.2), respectively. For example, sequelae of cerebrovascular disease (I69) with disorders of vestibular function (H81), chronic sinusitis (J32), cholelithiasis (K80), gonarthrosis (M17) or dorsopathies (M47, M50, and M51) tended to be more comorbid in males than in females, whereas kidney failure (N18, N19) with disorders of glycoprotein metabolism (E77) or disorders of purine and pyrimidine metabolism (E79) tended to be more comorbid in females than in males. Notably, the prevalence of other anxiety disorders (F41) in females (2.21%) was twice that of males (0.98%). In female IHD patients, other anxiety disorders were comorbid with sleep disorders (G47), cataracts (H25, H26), hypertension (I10, I11), cerebrovascular diseases (I63, I65, I67, I69), asthma (J45), diseases of the esophagus, stomach, and duodenum (K21, K25, K27, K29, K31), or dorsopathies (M47, M48, M50, M51) were statistically significant. Sex-specific comorbidity networks were divided into three distinct clusters for males and two distinct clusters for females. For age-specific comorbidity networks, the number of comorbidities varied little with age, but the mean number of nodules, with increasing network diameter, tended to increase. This was consistent with the increasing complexity of the relationship between comorbidities following the accumulation of chronic diseases. The prevalence of most physical disorders increased with age, while for mental disorders, such as other anxiety disorders (F41), these tended to decrease. The youngest age group (35–59 years) contained four different clusters; however, three clusters were obtained for all other age groups. All diseases included in the age- and sex-specific stratified clustering are shown in Supplementary Table S3.

## 4. Discussion

To the best of our knowledge, this is the first study in China to use a network-based approach and large-scale administrative data to systematically analyze the physical and mental comorbidity of patients hospitalized with IHD and compare these patients with matched controls. Analysis of comorbidity patterns may advance our understanding of associations between diseases rather than viewing them as a series of isolated illnesses. The results indicated that IHD inpatients showed 50% larger comorbidity burden when compared to controls. Most comorbidities, including physical and mental disorders, were more likely to co-occur with IHD (i.e., significant ORs after Bonferroni correction). Enormous and complex communities have been identified in the comorbidity network of IHD inpatients that aggregate comorbidities and comorbidity pairs of significance. Furthermore, the current study also reported differences in comorbidity prevalence, chronic disease coexistence, and community in sex and age sub-populations.

The inclusion of an entire spectrum of chronic diseases made it possible to investigate the chronic comorbidities of IHD in a comprehensive manner. Comorbidities in IHD inpatients were quite heterogeneous, with a few conditions exhibiting high prevalence and many others being less comorbid (e.g., 64.8% of comorbidities with a prevalence of 1% to 5%). Inpatients with IHD were twice as likely as controls to exhibit co-occurrence with 18 illnesses (ORs >2 with *p*-values < the Bonferroni corrected threshold), including diseases of the circular system (e.g., hypertension, heart failure, atrial fibrillation and flutter, cardiac arrhythmias, cardiomyopathy, and atherosclerosis), chronic renal failure, gastritis and duodenitis, and other metabolic diseases (e.g., diabetes mellitus, other hypothyroidism, and disorders of purine and pyrimidine metabolism). These are complex, system-level diseases that affect many physiological and biomolecular processes and therefore co-occur with many diseases. Meanwhile, negative associations of IHD with cataracts (H25, H26) and gonarthrosis (M17) were also found (Supplementary Table S1), which may be largely attributable to the inevitable selection bias in such retrospective studies. For example, people with only cataracts but not IHD are candidates for surgery and receive discharging diagnoses. Mental disorders have also been found to co-occur with IHD in previous studies [12,17,45]. In our study, the most significant differences among all mental comorbidities were observed in anxiety disorders, with the prevalence being twice that of controls. A greater burden of comorbidities among those with IHD was associated with a greater risk of death [9]. However, previous studies regarding IHD comorbidities were mostly based on statistics and restricted to single or a few diseases [10,11,12,13]. Therefore, a comprehensive assessment of the co-occurrence of physical and mental comorbidities is essential to better understand IHD, which facilitates the clinical management and medication used for treatment. 

Given the significant impact of cardiovascular diseases (CVDs) on human health, network-based approaches have been used in comorbidity studies of CVDs [20,24,25,46,47,48]. For example, a cross-sectional study on 34,099 discharged cases in Mexico found that comorbidity networks of CVDs were highly centralized in prevalent diseases, such as cardiac arrhythmias, heart failure, chronic kidney disease, hypertension, and ischemic diseases [20]. Another cross-sectional study in Italy found hypertension, arrhythmias, as well as kidney and lung diseases to be the disorders most associated with death, along with four clusters associated with cancer, lung disease, liver disease, and heart/circulation-related problems [47]. We found CVDs were not exclusive to IHD inpatients, but the prevalence and disease diversity of CVDs in IHD inpatients were higher than that observed in controls, which is in line with findings of the previous studies [4,6,7,8,9,10,49,50]. Interestingly, the connections between anxiety disorders (F41) and kinds of diseases (including sleep disturbance, cervical disc disorders, spondylosis, gastro-esophageal reflux disease, other functional intestinal disorders, disorders of vestibular function, cerebrovascular diseases, and atherosclerosis) were only observed in hospitalized patients with IHD, and had the largest OER = 7.10 (99% CI: 7.10–7.11). Comorbid anxiety alters other known risk factors (e.g., a sedentary lifestyle, substance use, and overweight), and also increases the risk of subsequent cardiac events and death in patients with IHD [51,52]. Anxiety seems to be under-recognized in its importance and is often not incorporated in subsequent prevention strategies in the routine clinical care of IHD, especially in developing countries. For example, the prevalence of under-diagnosed anxiety disorder was 5.3% in urban China, with only 0.5% of anxiety disorder respondents reporting a diagnosis [53]. Therefore, assessments of anxiety disorder during routine medical care should be performed to identify patients who would benefit from appropriate interventions.

Notably, the more densely connected comorbidities in IHD patients were not within the highly prevalent ones but the rarely prevalent ones. For instance, the top three most significant connections involving two disease systems were anxiety disorders (F41, prevalence = 1.62%) with sleep disorders (G47, prevalence = 2.03%), iron deficiency anemia (D50, prevalence = 1.18%) with gastric ulcer (K25, prevalence = 1.52%), and iron deficiency anemia (D50, prevalence = 1.18%) with peptic ulcer (site unspecified; K27, prevalence = 1.07%). Diseases can be related through several dimensions, such as sharing the same gene or the same pathway of proteins action, having the similar risk factors, or progressing along the same disease trajectory [29]. In our study, we identified the complex comorbidity profile in IHD patients by entire sets of chronic diseases, consisting of all highly and rarely prevalent ones, which might be helpful to understand some biological and medical questions from a perspective; this is complementary to other approaches. Additionally, a highly prevalent comorbidity in IHD patients was found with gastritis and duodenitis (K29), which had a higher average degree of neighbors in the case network than in control. Gastritis and duodenitis was more closely connected to other diseases, was associated with a higher prevalence of other diseases, and affected more diseases. A previous study showed that 81% of IHD patients had antral mucosal erosion and presented with significant erosive gastropathy accompanied by chronic inflammatory response activity in the duodenal mucosa [54]. Another study found that the occurrence of recurrent coronary phenomena within one year in patients with unstable angina and acute myocardial infarction was associated with high colonization of the gastric mucosa by Helicobacter pylori [55]. Based on the highly prevalent and highly connected comorbidities of digestive system diseases as mentioned above, attention should be focused on these digestive system diseases in IHD patients to better develop treatment plans and reduce medical burden. 

The modular partitioning of IHD patients gives rise to two large comorbidity clusters, compared to four clusters in the controls. The highly aggregated comorbidities in IHD patients may be attributed to their complex comorbidity relationships. Respiratory failure (not elsewhere classified; J96), hepatic failure (not elsewhere classified; K72), heart failure (I50), and kidney failure (N18, N19) are all complex syndromes responsible for high rates of death and hospitalization, which were clustered in the same community (Figure 4, Cluster A1). Multi-organ failure affects many physiological and biomolecular processes and therefore occurs with many diseases. In our study, we found that the comorbidity relationships between iron deficiency anemia (D50) and hepatic failure, not elsewhere classified (K72), and heart failure (I50) and kidney failure (N18, N19) were statistically significant. Given the undirected graph constructed, it is not possible to draw conclusions about the causality of the observed interactions. Further studies are needed to explore the disease progression trajectories within the cluster where multi-organ failure co-exists in IHD patients. Meanwhile, clusters often recapitulate physiopathological commonalities. Therefore, preferentially managing the common risk factors in each cluster should be a more efficient way of using limited resources to decrease comorbid burden. For example, cerebrovascular diseases (including ischemic stroke and sequelae) and cerebrovascular risk factors (hypertension, diabetes, and disorders of lipoprotein metabolism), as shown in Figure 4, Cluster A2. The Global Burden of Disease Study 2019 provides evidence that metabolic risk factors are the leading drivers of the burden of ischemic heart disease [56]. Hypertension alters the pathophysiology of ischemic stroke, such as vascular wall damage, blood–brain barrier disruption, hypoperfusion, and increased constriction [57].

Our results found that sex modified the comorbidity pattern in IHD patients. Overall, males had a higher comorbid burden than females. Furthermore, the PDN of males was more complex than that of females’ [58,59]. Physical disorders were generally higher in males than in females, while the opposite was true for the mental disorders found in our study (anxiety disorders, F41). This finding is consistent previous research by Mittendorfer-Rutz et al. (2018), who found that females were affected by common mental disorders (i.e., depressive and anxiety disorders) almost twice as often as males [60]. As reported by previous studies that women generally get IHD a few years later than men, which leads to a higher risk of comorbidities and work disability for females [60,61], and therefore leads to stressful and more mental comorbidities (e.g., anxiety disorders, sleep disorders). The male and female comorbidity clustering results showed that iron deficiency anemia (D50), chronic kidney failure (N18), and unspecified kidney failure (N19) were all clustered in the same group. Iron deficiency anemia is a vital complication in patients with advanced chronic kidney disease, as iron deficiency is one of the key mechanisms leading to erythropoiesis disorder in the case of renal dysfunction [62]. Additionally, 106 and 30 disease pairs involving two separate disease systems dominated in males and females. These sex-dominated comorbidity relationships provided enough information to develop sex-specific healthcare measures, i.e., the routine assessment of mental status for IHD patients in medical care, especially for females, was recommended; management of the common risk factors of CVDs should be a priority for male IHD patients. Moreover, sex-dominated comorbidity relationships might be useful to generate new hypotheses for sex differences in comorbidity patterns in IHD.

We also found that the comorbidity pattern in IHD patients was modified by age. With increasing age, IHD patients exhibited higher physical comorbid burden and the comorbidity network was more complicated. Aging accounted for the most comorbid burdens in IHD patients, which was similar to COPD patients, as some chronic diseases accumulate with age [63]. Meanwhile, the prevalence of mental diseases (e.g., anxiety disorders, F41) appears to decrease with age, with the greatest prevalence (2.63%) occurring in the middle age group (35–59 years). These results reflect those of Tran et al. (2018), who found that anxiety had the highest prevalence in those 40–49 years of age [48]. It may be that psychosocial adaptation to other life-threatening physical illnesses, such as cancer, is worse in younger people and is more likely to interfere with daily and working life than in older age groups [17]. The central diseases in the middle age group (35–59 years) of IHD were less severe than those of older age group (60–69 years). Therefore, in order to prevent the occurrence of further comorbidity relationships, delay the progression of the disease to a serious state and improve the quality of life, it is recommended that the management of middle age group (35–59 years) IHD patients might be prioritized for disorders resulting from impaired kidney tubular function, menopausal and other perimenopausal disorders, cerebral infarction, other pulmonary heart diseases, and mental disorders in order to prevent further comorbidities.

Some limitations to our study should be mentioned. First, given the undirected weighted graph constructed, it is not possible to draw conclusions about the causality and interactions of the observed associations. Second, we excluded individuals who died during the study period to obtain a more homogenous sample, which may underestimate the comorbidity complexity. Although previous studies have indicated that this excluding criteria did not drastically affect the results [29,64], it is vital to interpret our findings in the context of the living inpatient population. Finally, some comorbidities may have not been identified, possibly resulting in an underestimation of the complexity of the comorbidity network. For instance, obesity is generally diagnosed when patients need medication or surgical treatment in the Chinese population (<1% prevalence in both IHD patients and controls) and thus, is largely underestimated at the population level [65,66]. Therefore, it is important to interpret our results in the context of a population of hospitalized patients for IHD in China.

Although the above-mentioned limitations are common in retrospective studies using administrative data, this study has several strengths. First, the present study included a large and representative sample of Chinese populations which provided a unique opportunity to study the entire spectrum of phenotypic chronic diseases rather than focusing on a single disease or a small number of them [9,10,11,12,13]. Second, using network theory and diagnostic codes from discharge data, a more in-depth exploration of the complex relationships between chronic conditions was undertaken by obtaining a profile of significant comorbidities in IHD inpatients [67]. Last, to the best of our knowledge, this is the first case–control study in China to investigate differences in comorbidity status between IHD and non-IHD inpatients, which has important implications for the integrated management of inpatients with IHD [68].

In terms of directions for future research, further work could incorporate more types of data, such as medical images and quantitative features, and construct directed comorbidity networks [69] or patient networks [70] to generate domain knowledge, then extract features from the networks and identify the knowledge embedded in the networks through machine learning algorithms [71,72], so as to effectively predict the health risk of patients.

## 5. Conclusions

In summary, our systematic analysis of comorbidities in hospitalized patients with IHD in China provides an overview of chronic physical and mental comorbidity of IHD in routine inpatient care. IHD inpatients showed 50% larger comorbid burden than the matched controls, and thus more complex comorbidity relationships, especially in males and older IHD patients. In addition, we detected sex-dominated co-occurrence disease pairs and highly inter-linked communities in IHD patients, which provides a new insight into the comorbidity pattern. The data-driven discovery of comorbid disease pairs may support clinicians and patients in making more specific diagnoses and treatment plans by considering the patients’ sex, age, and comorbidities.

## Figures and Tables

**Table 1:** Prevalence of the top 20 comorbid chronic diseases in cases and their ORs (95% CI).

Chronic Disease	ICD-10	Prevalence (%, 95% CI)		OR (95% CI)
		IHD Inpatients	Controls	
Other anemias	D64	7.16 (7.11–7.21)	6.19 (6.15–6.24)	1.17 (1.16–1.18) *
Diabetes mellitus	E11	20.09 (20.01–20.17)	10.75 (10.69–10.81)	2.09 (2.07–2.10) *
Disorders of lipoprotein metabolism and other lipidemias	E78	15.71 (15.64–15.78)	8.59 (8.53–8.64)	1.98 (1.97–2.00) *
Hypertension	I10	48.64 (48.54–48.73)	29.98 (29.90–30.07)	2.21 (2.20–2.22) *
Other pulmonary heart diseases	I27	7.86 (7.81–7.91)	5.61 (5.57–5.66)	1.43 (1.42–1.45) *
Atrial fibrillation and flutter	I48	7.72 (7.66–7.77)	1.90 (1.87–1.92)	4.33 (4.26–4.40) *
Other cardiac arrhythmias	I49	8.86 (8.81–8.92)	2.94 (2.90–2.97)	3.22 (3.17–3.26) *
Heart failure	I50	29.39 (29.30–29.48)	4.93 (4.89–4.97)	8.02 (7.94–8.10) *
Complications and ill-defined descriptions of heart disease	I51	8.32 (8.27–8.38)	2.32 (2.29–2.35)	3.82 (3.77–3.88) *
Cerebral infarction	I63	19.92 (19.84–20.00)	13.91 (13.84–13.97)	1.54 (1.53–1.55) *
Other cerebrovascular diseases	I67	15.90 (15.83–15.97)	8.80 (8.75–8.86)	1.96 (1.94–1.97) *
Atherosclerosis	I70	14.66 (14.59–14.72)	7.24 (7.19–7.29)	2.20 (2.18–2.22) *
Other chronic obstructive pulmonary disease	J44	23.45 (23.36–23.53)	15.55 (15.49–15.62)	1.66 (1.65–1.67) *
Gastritis and duodenitis	K29	29.10 (29.01–29.18)	16.15 (16.07–16.22)	2.13 (2.12–2.15) *
Other diseases of liver	K76	12.14 (12.08–12.20)	8.46 (8.41–8.51)	1.50 (1.48–1.51) *
Cholelithiasis	K80	7.35 (7.30–7.40)	7.29 (7.24–7.34)	1.01 (1.00–1.02)
Spondylosis	M47	7.26 (7.21–7.31)	5.23 (5.19–5.28)	1.42 (1.40–1.43) *
Other intervertebral disc disorders	M51	7.52 (7.47–7.57)	7.40 (7.35–7.45)	1.02 (1.01–1.03) *
Other disorders of kidney and ureter, not elsewhere classified	N28	8.53 (8.48–8.59)	6.99 (6.94–7.04)	1.24 (1.23–1.25) *
Hyperplasia of prostate	N40	20.82 (20.70–20.93)	7.92 (7.87–7.97)	1.30 (1.29–1.31) *

ORs: odds ratios; CI: confidence interval; *: statistically significant ORs after Bonferroni correction.

**Table 2:** Metrics of PDNs in IHD patients and controls.

Metrics	Case	Control	*p*-Value
No. of patients	1,035,338	1,035,338	N/A
Nodes	71	63	N/A
Edges	1941	1372	N/A
Diameter ^1^	2.44	3.42	N/A
Avg. Degree ^2^	54.68	43.56	<0.0001
Avg. Closeness ^3^	0.83	0.78	0.0138
Avg. Neighbor. Degree ^4^	55.88	46.53	<0.0001

^1^ Diameter: the maximum value of the weighted distance between any two nodes in the network; ^2^ Avg. Degree: average number of links of all nodes in the network to other nodes; ^3^ Avg. Closeness: the average of the inverse of sum of the shortest path lengths between the disease and all other diseases in the graph; ^4^ Avg. Neighbor. Degree: the average degree of neighbors for each node.

**Table 3:** Metrics of sex- and age-specific PDNs.

Metrics	Sex			Age Group				
	Male	Female	*p*-Value	35–59	60–69	70–79	80+	*p*-Value
No. of patients	500,785	534,553	N/A	139,462	287,320	394,282	214,274	N/A
Nodes	72	67	N/A	74	70	74	71	N/A
Edges	2019	1683	N/A	1685	1696	2090	2052	N/A
Diameter	2.61	2.52	N/A	3.25	2.46	2.52	2.50	N/A
Avg. Degree	56.08	50.24	<0.0001	45.54	48.46	56.49	57.80	<0.0001
Avg. Closeness	0.84	0.82	0.0009	0.73	0.78	0.82	0.86	<0.0001
Avg. Neighbor. Degree	57.71	51.51	<0.0001	46.67	50.08	58.08	59.03	<0.0001

## Data Availability

Due to ethical restrictions, the data that support the findings of this study are not publicly available.

## Supplementary Materials

The following supporting information can be downloaded at: https://www.mdpi.com/article/10.3390/healthcare10010080/s1, Phenotypic Disease Network Analysis to Identify Comorbidity Patterns in Hospitalized Patients with Ischemic Heart Disease Using Large-Scale Administrative Data.
