# Supplementary material for: Phenotypic Disease Network Analysis to Identify Comorbidity Patterns in Hospitalized Patients with Ischemic Heart Disease Using Large-Scale Administrative Data
Source: Healthcare (Basel). 2022 Jan 1;10(1):80. doi: 10.3390/healthcare10010080 (PMC8775672; doi:10.3390/healthcare10010080)
Supplement: Supplementary file 1 [file healthcare-10-00080-s001.zip › healthcare-1524272-supplementary.pdf]

Supplementary Materials

Phenotypic Disease Network Analysis to Identify Comorbidity Patterns in Hospitalized Patients with Ischemic Heart Disease Using Large-Scale Administrative Data

Dejia Zhou [1,2,†], Liya Wang [1,†], Shuhan Ding [3], Minghui Shen [4] and Hang Qiu [1,2,*]

1 Big Data Research Center, University of Electronic Science and Technology of China, Chengdu 611731, China; zhoudj@std.uestc.edu.cn (D.Z.); hbigdata@uestc.edu.cn (L.W.); qiuhang@uestc.edu.cn (H.Q.)

2 School of Computer Science and Engineering, University of Electronic Science and Technology of China, Chengdu 611731, China;

3 School of Electrical and Computer Engineering, Cornell University, Ithaca, NY 14853, USA; sd925@cornell.edu

4 Health Information Center of Sichuan Province, Chengdu 610041, China; Shenmh@schnic.cn

*** Correspondence: qiuhang@uestc.edu.cn (H.Q.); Tel.: 86-28-61830278**

† These authors contributed equally to this work.

Contents

1 Data set 1
2 Identifying comorbidities 1
3 Quantifying Comorbidity Strength..4
4 Comorbidity network and clusters in IHD patients 5
5 Sex- and Age-specific comorbidity networks 20
6 References 22

1 Data set

We used a provincial healthcare database between 2015 and 2019 where the reasons for all hospitalizations were reported in ICD-10-CM format. Each hospital discharge record consists of a unique patient identifier, sex, age, date of admission and discharge, hospitalization expenses, primary discharge diagnosis and up to 15 secondary diagnoses.

Totally, 1,035,338 IHD inpatients, living in Sichuan Province, aged ≥ 35 years, with a diagnosis of I20-I25 and alive during the entire study period, were included. For each IHD inpatient, a one-to-one matched control was randomly selected from inpatients without IHD during the entire study period and individually matched to the index patient by year of birth (± 2 years), sex, discharge date and level of hospital.

To investigate the demographic difference in comorbidity patterns in IHD, we distinguish two main groups in the data set given by sex (male and female) and age (35-59, 60-69, 70-79 and ≥ 80 years), as shown in Figure S1.

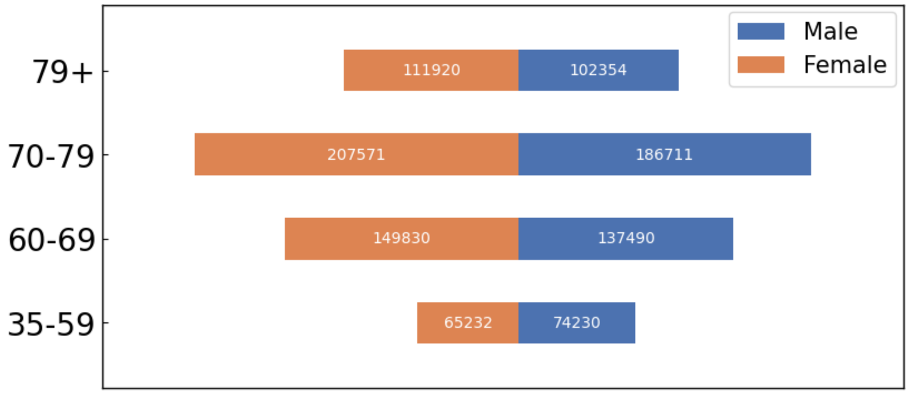

Figure S1. Age group distribution for IHD patients by gender.

2 Identifying comorbidities

2.1 Prevalence of chronic conditions in IHD patients and controls

For both IHD patients and controls, diagnoses were assessed from the hospital discharge records during the study period. We examined all diagnoses using the ICD-10 codes at three digits and applied the Chronic Condition Indicator to differentiate between acute and chronic diseases [1,2]. We excluded diagnoses from chapters XIX (injury, poisoning, and certain other consequences of external causes), chapters XX (external causes of morbidity) and chapters XXI (factors influencing health status and contact with health services), since they are not diseases or just general symptoms. We further excluded rarely occurred (prevalence < 1%) chronic conditions. Finally, 71 and 63 chronic diseases were included and their prevalence are shown in Table S1.

OR is the odds ratio of having a comorbidity, comparing IHD patients against controls, calculated using chi-square statistic. Bonferroni correction was applied to correct for multiple hypotheses testing; a p-value of less than 0.05/71 was considered statistically significant.

Table S1. Prevalence of chronic conditions in IHD patients and controls.

Chronic disease	ICD-10	Prevalence (%, 95% CI[1])		OR[2] (95% CI)
		IHD patients	controls	
Hypertension	I10	48.64(48.54-48.73)	29.98(29.90-30.07)	2.21(2.20-2.22)*
Heart failure	I50	29.39(29.30-29.48)	4.93(4.89-4.97)	8.02(7.94-8.10)*
Gastritis and duodenitis	K29	29.10(29.01-29.18)	16.15(16.07-16.22)	2.13(2.12-2.15)*
Other chronic obstructive pulmonary disease	J44	23.45(23.36-23.53)	15.55(15.49-15.62)	1.66(1.65-1.67)*
Hyperplasia of prostate	N40	20.82(20.70-20.93)	7.92(7.87-7.97)	1.30(1.29-1.31)*
Diabetes mellitus	E11	20.09(20.01-20.17)	10.75(10.69-10.81)	2.09(2.07-2.10)*
Cerebral infarction	I63	19.92(19.84-20.00)	13.91(13.84-13.97)	1.54(1.53-1.55)*
Other cerebrovascular diseases	I67	15.90(15.83-15.97)	8.80(8.75-8.86)	1.96(1.94-1.97)*
Disorders of lipoprotein metabolism and other lipidaemias	E78	15.71(15.64-15.78)	8.59(8.53-8.64)	1.98(1.97-2.00)*
Atherosclerosis	I70	14.66(14.59-14.72)	7.24(7.19-7.29)	2.20(2.18-2.22)*
Other diseases of liver	K76	12.14(12.08-12.20)	8.46(8.41-8.51)	1.50(1.48-1.51)*
Other cardiac arrhythmias	I49	8.86(8.81-8.92)	2.94(2.90-2.97)	3.22(3.17-3.26)*
Other disorders of kidney and ureter, not elsewhere classified	N28	8.53(8.48-8.59)	6.99(6.94-7.04)	1.24(1.23-1.25)*
Complications and ill-defined descriptions of heart disease	I51	8.32(8.27-8.38)	2.32(2.29-2.35)	3.82(3.77-3.88)*
Other pulmonary heart diseases	I27	7.86(7.81-7.91)	5.61(5.57-5.66)	1.43(1.42-1.45)*
Atrial fibrillation and flutter	I48	7.72(7.66-7.77)	1.90(1.87-1.92)	4.33(4.26-4.40)*
Other intervertebral disc disorders	M51	7.52(7.47-7.57)	7.40(7.35-7.45)	1.02(1.01-1.03)
Cholelithiasis	K80	7.35(7.30-7.40)	7.29(7.24-7.34)	1.01(1.00-1.02)
Spondylosis	M47	7.26(7.21-7.31)	5.23(5.19-5.28)	1.42(1.40-1.43)*
Other anaemias	D64	7.16(7.11-7.21)	6.19(6.15-6.24)	1.17(1.16-1.18)*
Transient cerebral ischaemic attacks and related syndromes	G45	7.15(7.10-7.20)	4.80(4.76-4.84)	1.53(1.51-1.55)*
Hypertensive heart disease	I11	6.91(6.86-6.96)	3.07(3.03-3.10)	2.35(2.31-2.38)*
Pneumonia, organism unspecified	J18	5.84(5.80-5.89)	4.07(4.03-4.11)	1.46(1.44-1.48)*
Unspecified chronic bronchitis	J42	5.62(5.58-5.67)	3.75(3.71-3.78)	1.53(1.51-1.55)*
Osteoporosis without pathological fracture	M81	5.29(5.25-5.34)	4.62(4.58-4.66)	1.15(1.14-1.17)*
Other spondylopathies	M48	4.93(4.89-4.98)	4.26(4.22-4.30)	1.17(1.15-1.18)*
Disorders of purine and pyrimidine metabolism	E79	4.87(4.83-4.91)	2.24(2.21-2.27)	2.24(2.20-2.27)*
Disorders of glycoprotein metabolism	E77	4.87(4.83-4.91)	4.90(4.86-4.94)	0.99(0.98-1.01)
Emphysema	J43	4.78(4.74-4.82)	3.53(3.50-3.57)	1.37(1.35-1.39)*
Other degenerative diseases of nervous system	G31	4.74(4.70-4.78)	3.54(3.51-3.58)	1.35(1.34-1.37)*
Respiratory failure, not elsewhere classified	J96	3.64(3.60-3.67)	2.60(2.57-2.63)	1.41(1.39-1.44)*
Chronic kidney failure	N18	3.60(3.56-3.63)	1.56(1.53-1.58)	2.36(2.31-2.40)*
Sequelae of cerebrovascular disease	I69	3.46(3.42-3.49)	2.07(2.04-2.10)	1.69(1.66-1.72)*
Other disorders of urinary system	N39	3.14(3.11-3.18)	3.19(3.16-3.22)	0.99(0.97-1.00)
Other noninfective gastroenteritis and colitis	K52	2.93(2.89-2.96)	2.32(2.29-2.34)	1.27(1.25-1.29)*
Occlusion and stenosis of precerebral arteries	I65	2.82(2.79-2.85)	1.39(1.37-1.42)	2.05(2.01-2.10)*
Unspecified diabetes mellitus	E14	2.76(2.72-2.79)	1.58(1.56-1.61)	1.76(1.73-1.79)*
Senile cataract	H25	2.53(2.50-2.56)	4.84(4.80-4.88)	0.51(0.50-0.52)*
Other diseases of stomach and duodenum	K31	2.43(2.40-2.46)	2.01(1.98-2.04)	1.22(1.19-1.24)*
Cervical disc disorders	M50	2.38(2.35-2.41)	1.79(1.77-1.82)	1.34(1.31-1.36)*
Hepatic failure, not elsewhere classified	K72	2.31(2.29-2.34)	1.92(1.90-1.95)	1.21(1.19-1.23)*
Bronchiectasis	J47	2.20(2.17-2.23)	1.78(1.75-1.81)	1.24(1.22-1.27)*
Gonarthrosis [arthrosis of knee]	M17	2.12(2.09-2.15)	2.19(2.17-2.22)	0.96(0.95-0.98)*
Gastro-oesophageal reflux disease	K21	2.06(2.03-2.09)	1.08(1.06-1.10)	1.94(1.89-1.98)*

Sleep disorders	G47	2.03(2.01-2.06)	1.07(1.05-1.09)	1.92(1.87-1.96)*
Gout	M10	2.01(1.99-2.04)	1.05(1.03-1.07)	1.94(1.90-1.99)*
Other functional intestinal disorders	K59	1.97(1.94-2.00)	1.52(1.49-1.54)	1.31(1.28-1.33)*
Disorders of vestibular function	H81	1.78(1.75-1.80)	1.48(1.46-1.50)	1.20(1.18-1.23)*
Endocarditis, valve unspecified	I38	1.74(1.71-1.76)	1.05(1.03-1.07)	1.66(1.62-1.70)*
Purpura and other haemorrhagic conditions	D69	1.72(1.70-1.75)	1.56(1.54-1.58)	1.11(1.08-1.13)*
Unspecified kidney failure	N19	1.66(1.64-1.69)	0.90(0.88-0.92)	1.86(1.81-1.91)*
Other disorders of bone	M89	1.63(1.61-1.66)	1.37(1.35-1.40)	1.19(1.16-1.22)*
Atrioventricular and left bundle-branch block	I44	1.63(1.60-1.65)	0.56(0.54-0.57)	2.96(2.87-3.05)*
Other anxiety disorders	F41	1.62(1.59-1.64)	0.83(0.82-0.85)	1.96(1.91-2.01)*
Other hypothyroidism	E03	1.53(1.51-1.55)	0.69(0.67-0.70)	2.25(2.19-2.31)*
Gastric ulcer	K25	1.52(1.50-1.55)	1.25(1.23-1.27)	1.22(1.19-1.25)*
Paroxysmal tachycardia	I47	1.52(1.49-1.54)	0.42(0.41-0.43)	3.63(3.51-3.76)*
Other diseases of gallbladder	K82	1.52(1.49-1.54)	1.26(1.24-1.28)	1.21(1.18-1.24)*
Chronic sinusitis	J32	1.51(1.49-1.54)	1.48(1.46-1.50)	1.02(1.00-1.05)
Other nontoxic goitre	E04	1.38(1.35-1.40)	1.03(1.01-1.05)	1.34(1.31-1.38)*
Asthma	J45	1.29(1.27-1.31)	0.86(0.84-0.87)	1.52(1.48-1.56)*
Other diseases of pericardium	I31	1.29(1.27-1.31)	0.58(0.56-0.59)	2.25(2.18-2.32)*
Other cataract	H26	1.23(1.21-1.25)	1.57(1.55-1.59)	0.78(0.76-0.80)*
Other rheumatoid arthritis	M06	1.21(1.19-1.23)	0.87(0.86-0.89)	1.38(1.35-1.42)*
Other headache syndromes	G44	1.20(1.18-1.22)	0.75(0.73-0.76)	1.61(1.56-1.65)*
Iron deficiency anaemia	D50	1.18(1.16-1.20)	1.07(1.05-1.09)	1.10(1.07-1.13)
Cardiomyopathy	I42	1.12(1.10-1.14)	0.45(0.44-0.46)	2.51(2.43-2.60)*
Abnormal findings on diagnostic imaging of other body structures	R93	1.11(1.09-1.13)	0.98(0.96-1.00)	1.14(1.11-1.17)*
Other conduction disorders	I45	1.10(1.08-1.12)	0.49(0.48-0.51)	2.26(2.18-2.33)*
Peptic ulcer, site unspecified	K27	1.07(1.05-1.09)	0.71(0.69-0.73)	1.51(1.47-1.56)*
Other interstitial pulmonary diseases	J84	1.01(0.99-1.03)	0.64(0.62-0.66)	1.58(1.53-1.63)*

[1] CI: confidence interval; [2]OR: odds ratio; *: statistical significance of ORs after Bonferroni correction.

2.2 Odds ratio for comorbidities

Scatter plot depicting ORs for comorbidities and their prevalence estimates is shown in Figure S2.

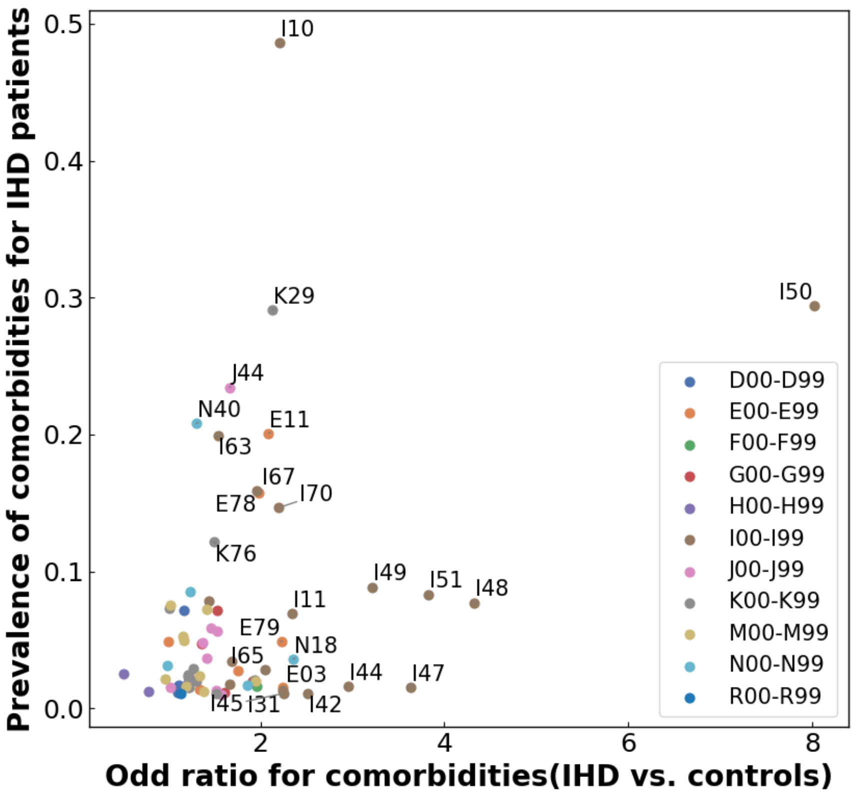

Figure S2. Scatter plot depicting odds ratios for comorbid conditions in case over their prevalence estimates.

3 Quantifying Comorbidity Strength

The relative risk is a measure of comorbidity strength used in previous studies of disease networks [3–6]. The relative risk used in disease networks often refers to the observed-to-expected ratio (OER). As the nomenclature "RR" is conventionally used in epidemiology and biostatistics, we denote the relative risk in our comorbidity network by OER rather than RR to minimize the confusion. The observed prevalence (O) of a pair of diseases i and j can be expressed mathematically as:

$$O_{ij} = \frac{C_{ij}}{N}$$

If a pair of diseases i and j occurred independently, the expected prevalence (E) of the disease-pair based on the product of disease i prevalence and disease j prevalence would be given by:

$$E_{ij} = \frac{C_i}{N} * \frac{C_j}{N}$$

Hence the OER of a pair of diseases is given by:

$$OER_{ij} = \frac{O_{ij}}{E_{ij}} = \frac{C_{ij}N}{C_iC_j}$$

where Cij is the number of patients co-existence with both diseases, N is the total number of patients in the population, and Ci and Cj are the number of patients diagnosed with diseases i and j.

Calculating the significance of the OER can be done by estimating confidence intervals [7]. In our study, we calculated the 99% confidence interval (99% CI) for the OER between diseases i and j is given by:

$$OER_{ij} * exp(\pm 2.58\sigma_{ij}), \text{ with}$$

$$\sigma_{ij} = \frac{1}{C_{ij}} + \frac{1}{C_i C_j} - \frac{1}{N} - \frac{1}{N^2}$$

4 Comorbidity network and clusters in IHD patients

Extensive comorbidity data are provided in the form of a comorbidity network for IHD patients, as shown in Table S2. Table S3 shows the distinct clusters in the network of IHD patients and controls.

Table S2. Data in the form of comorbidity network in IHD patients.

ID	Disease pair	OER (99% CI)	ID	Disease pair	OER (99% CI)	ID	Disease pair	OER (99% CI)
1	I44,I45	8.5 (8.49, 8.51)	48	G44,H81	3.55 (3.54, 3.56)	95	J32,M50	2.84 (2.84, 2.85)
2	F41,G47	7.1 (7.1, 7.11)	49	J43,J47	3.55 (3.54, 3.55)	96	E04,M50	2.84 (2.83, 2.85)
3	D50,K25	6.76 (6.74, 6.77)	50	J84,M06	3.54 (3.52, 3.56)	97	E77,N18	2.82 (2.82, 2.82)
4	D50,K27	6.56 (6.54, 6.58)	51	M50,M51	3.53 (3.53, 3.53)	98	I31,K72	2.79 (2.78, 2.8)
5	N18,N19	6.3 (6.3, 6.3)	52	E77,N19	3.51 (3.51, 3.51)	99	J43,J96	2.79 (2.79, 2.79)
6	I27,J96	6.2 (6.2, 6.2)	53	E79,N18	3.49 (3.49, 3.5)	100	I27,J43	2.79 (2.78, 2.79)
7	I45,I47	6.14 (6.13, 6.16)	54	M06,M17	3.41 (3.4, 3.42)	101	I38,I48	2.78 (2.78, 2.78)
8	M10,N18	5.53 (5.53, 5.53)	55	I42,I44	3.35 (3.33, 3.36)	102	F41,H81	2.78 (2.77, 2.79)
9	J47,J96	5.34 (5.33, 5.34)	56	J42,J43	3.32 (3.32, 3.32)	103	E04,M47	2.77 (2.77, 2.77)
10	F41,G44	5.12 (5.11, 5.13)	57	M48,M81	3.32 (3.32, 3.32)	104	J44,J47	2.77 (2.77, 2.77)
11	D64,N18	4.96 (4.96, 4.96)	58	J18,J96	3.27 (3.27, 3.28)	105	G47,K21	2.76 (2.76, 2.77)
12	M17,M81	4.92 (4.92, 4.93)	59	J44,J96	3.27 (3.27, 3.27)	106	I42,N19	2.76 (2.74, 2.77)
13	M17,M51	4.81 (4.8, 4.81)	60	E79,I42	3.23 (3.23, 3.24)	107	K76,N28	2.75 (2.75, 2.75)
14	H25,H26	4.72 (4.71, 4.73)	61	H81,M50	3.21 (3.21, 3.22)	108	D50,N18	2.75 (2.74, 2.75)
15	G47,K59	4.7 (4.7, 4.71)	62	I42,I48	3.21 (3.2, 3.21)	109	G44,G45	2.68 (2.68, 2.69)
16	E04,M17	4.69 (4.69, 4.7)	63	I48,I51	3.2 (3.2, 3.2)	110	F41,K59	2.68 (2.68, 2.69)
17	D64,E77	4.66 (4.66, 4.66)	64	K21,K31	3.17 (3.16, 3.17)	111	I27,J84	2.67 (2.67, 2.68)
18	D50,D64	4.45 (4.45, 4.45)	65	I27,J45	3.12 (3.12, 3.12)	112	I50,I51	2.65 (2.65, 2.65)
19	I47,I49	4.4 (4.4, 4.4)	66	E77,I31	3.1 (3.1, 3.11)	113	I31,I51	2.65 (2.65, 2.65)
20	E03,E04	4.31 (4.29, 4.32)	67	E04,G47	3.1 (3.09, 3.11)	114	M47,M50	2.64 (2.64, 2.65)
21	D64,N19	4.23 (4.23, 4.24)	68	D69,K72	3.09 (3.08, 3.1)	115	K82,N28	2.64 (2.64, 2.64)
22	G31,J32	4.2 (4.2, 4.2)	69	I45,I49	3.09 (3.08, 3.09)	116	I44,I49	2.64 (2.64, 2.64)
23	E77,K72	4.14 (4.14, 4.14)	70	D64,D69	3.08 (3.08, 3.08)	117	D64,R93	2.03 (2.02, 2.03)
24	M10,N19	4.14 (4.13, 4.14)	71	I42,K72	3.07 (3.06, 3.08)	118	I38,I51	2.63 (2.62, 2.63)
25	I42,I51	4.13 (4.13, 4.13)	72	M17,M48	3.07 (3.07, 3.07)	119	E11,E14	2.61 (2.61, 2.61)
26	J45,J96	4.06 (4.05, 4.06)	73	K25,K31	3.06 (3.06, 3.07)	120	I44,I47	2.6 (2.59, 2.61)
27	J84,J96	4.04 (4.03, 4.04)	74	F41,M50	3.06 (3.06, 3.07)	121	I11,N18	2.6 (2.59, 2.6)
28	J43,J84	4.03 (4.02, 4.03)	75	D64,K27	3.05 (3.05, 3.05)	122	M17,M50	2.59 (2.59, 2.6)
29	I31,I42	4.01 (3.99, 4.03)	76	J96,K72	3.04 (3.04, 3.04)	123	N28,N40	2.59 (2.59, 2.59)
30	J47,J84	3.99 (3.98, 4)	77	G44,J32	3 (2.98, 3.01)	124	H81,M47	2.58 (2.57, 2.58)
31	E79,N19	3.98 (3.98, 3.98)	78	D64,K25	2.99 (2.99, 2.99)	125	D69,R93	1.87 (1.85, 1.88)
32	I27,J47	3.98 (3.98, 3.98)	79	E14,H26	2.99 (2.98, 3)	126	M81,M89	2.57 (2.57, 2.57)
33	M51,M89	3.91 (3.91, 3.91)	80	M17,M47	2.96 (2.96, 2.96)	127	E77,J84	2.57 (2.56, 2.57)
34	K72,N19	3.9 (3.89, 3.9)	81	I31,I38	2.96 (2.95, 2.97)	128	K27,M06	2.55 (2.53, 2.56)
35	M51,M81	3.89 (3.89, 3.89)	82	F41,K21	2.95 (2.95, 2.96)	129	D64,M06	2.53 (2.53, 2.54)
36	E77,J96	3.88 (3.88, 3.88)	83	K52,K59	2.95 (2.95, 2.96)	130	G44,M50	2.51 (2.5, 2.52)
37	M48,M51	3.81 (3.81, 3.82)	84	D50,N19	2.94 (2.93, 2.96)	131	K76,K82	2.51 (2.51, 2.51)
38	I27,J44	3.79 (3.79, 3.79)	85	D50,R93	2.27 (2.25, 2.29)	132	I31,N19	2.51 (2.49, 2.52)
39	K25,K27	3.72 (3.71, 3.74)	86	F41,G45	2.94 (2.94, 2.94)	133	E77,K27	2.5 (2.5, 2.51)
40	M48,M50	3.72 (3.72, 3.72)	87	G45,M50	2.91 (2.91, 2.92)	134	E77,J18	2.5 (2.5, 2.5)
41	J45,J47	3.72 (3.71, 3.73)	88	G31,I63	2.91 (2.91, 2.91)	135	E04,M81	2.5 (2.49, 2.5)
42	M50,M89	3.69 (3.68, 3.7)	89	M47,M51	2.9 (2.9, 2.91)	136	G47,M50	2.49 (2.48, 2.49)
43	G44,G47	3.69 (3.68, 3.7)	90	K25,M06	2.89 (2.88, 2.91)	137	D69,N19	2.48 (2.47, 2.49)
44	I42,I47	3.67 (3.65, 3.68)	91	K21,K59	2.88 (2.88, 2.89)	138	D50,M06	2.47 (2.46, 2.49)
45	E79,M10	3.66 (3.66, 3.66)	92	E04,F41	2.88 (2.87, 2.89)	139	K21,K25	2.47 (2.47, 2.48)
46	D50,E77	3.6 (3.59, 3.6)	93	E03,I31	2.87 (2.86, 2.88)	140	I42,I45	2.46 (2.44, 2.48)
47	M06,M81	3.58 (3.57, 3.58)	94	D69,E77	2.85 (2.85, 2.86)	141	I31,I48	2.43 (2.42, 2.43)

TABLE S2. Data in the form of comorbidity network in IHD patients (continued)

ID	Disease pair	OER (99% CI)	ID	Disease pair	OER (99% CI)	ID	Disease pair	OER (99% CI)
142	E77,K59	2.42 (2.42, 2.43)	189	I44,I51	2.21 (2.21, 2.21)	236	J18,J84	2.06 (2.05, 2.06)
143	E04,I70	2.42 (2.42, 2.42)	190	K27,K31	2.2 (2.19, 2.21)	237	G47,I67	2.05 (2.05, 2.05)
144	K25,M10	2.42 (2.41, 2.43)	191	E77,J47	2.2 (2.2, 2.21)	238	I69,K59	2.04 (2.04, 2.05)
145	E04,K76	2.41 (2.41, 2.41)	192	M17,M89	2.2 (2.2, 2.21)	239	E11,N18	2.04 (2.04, 2.04)
146	I11,I51	2.41 (2.41, 2.41)	193	J32,M89	2.2 (2.19, 2.21)	240	E11,H26	2.04 (2.04, 2.04)
147	G31,M50	2.41 (2.41, 2.41)	194	I11,M10	2.19 (2.19, 2.2)	241	F41,K31	2.03 (2.03, 2.04)
148	G47,H81	2.39 (2.38, 2.4)	195	E78,E79	2.19 (2.19, 2.19)	242	G31,R93	1.81 (1.81, 1.82)
149	I27,I31	2.39 (2.39, 2.39)	196	N39,N40	2.19 (2.19, 2.19)	243	H81,J32	2.02 (2.01, 2.03)
150	D64,I31	2.38 (2.38, 2.39)	197	E04,K21	2.18 (2.18, 2.19)	244	E77,K25	2.02 (2.02, 2.03)
151	E77,M06	2.38 (2.38, 2.39)	198	E14,N19	2.18 (2.18, 2.19)	245	E78,G47	2.02 (2.02, 2.02)
152	E04,J32	2.38 (2.37, 2.39)	199	E03,F41	2.17 (2.16, 2.18)	246	G47,I11	2.02 (2.01, 2.02)
153	K72,K80	2.38 (2.38, 2.38)	200	E14,R93	1.02 (1.01, 1.02)	247	I63,I65	2.02 (2.01, 2.02)
154	I31,J96	2.38 (2.37, 2.38)	201	E03,G47	2.17 (2.16, 2.18)	248	D50,I31	2.01 (2, 2.03)
155	M48,M89	2.37 (2.37, 2.38)	202	E77,R93	2.36 (2.35, 2.36)	249	I38,I49	2 (2, 2.01)
156	E79,K72	2.37 (2.37, 2.37)	203	E78,R93	1.47 (1.47, 1.47)	250	I42,N18	2 (2, 2.01)
157	K59,N39	2.37 (2.36, 2.37)	204	F41,M47	2.16 (2.16, 2.16)	251	I38,I44	2 (1.99, 2.01)
158	E04,K82	2.36 (2.35, 2.38)	205	J45,K21	2.16 (2.15, 2.17)	252	G44,M47	2 (2, 2)
159	E03,R93	1.38 (1.36, 1.39)	206	I67,M50	2.15 (2.15, 2.15)	253	H26,N18	1.99 (1.99, 2)
160	I38,I42	2.36 (2.35, 2.37)	207	E04,M48	2.15 (2.15, 2.15)	254	G31,M48	1.99 (1.98, 1.99)
161	J44,J45	2.36 (2.36, 2.36)	208	G44,I67	2.14 (2.14, 2.14)	255	G31,M89	1.98 (1.98, 1.99)
162	M10,M17	2.35 (2.35, 2.36)	209	J32,M47	2.13 (2.13, 2.14)	256	G31,K59	1.98 (1.98, 1.99)
163	E03,N18	2.35 (2.35, 2.35)	210	K31,K59	2.13 (2.13, 2.14)	257	G44,R93	1.41 (1.39, 1.43)
164	N19,N39	2.35 (2.34, 2.35)	211	E77,E79	2.13 (2.13, 2.13)	258	I31,J43	1.98 (1.98, 1.98)
165	J96,N19	2.34 (2.34, 2.35)	212	J18,J47	2.13 (2.13, 2.13)	259	I67,M47	1.98 (1.98, 1.98)
166	I42,I50	2.33 (2.33, 2.34)	213	M47,M48	2.13 (2.13, 2.13)	260	K21,K52	1.98 (1.97, 1.98)
167	G31,I69	2.33 (2.33, 2.33)	214	E79,I11	2.13 (2.13, 2.13)	261	E04,K59	1.97 (1.96, 1.98)
168	D50,M10	2.33 (2.32, 2.34)	215	E04,H26	2.12 (2.11, 2.14)	262	E04,J45	1.97 (1.95, 1.98)
169	D50,K31	2.32 (2.32, 2.33)	216	I63,I69	2.11 (2.11, 2.11)	263	I38,N19	1.96 (1.95, 1.97)
170	E04,R93	2.63 (2.61, 2.64)	217	K59,M81	2.11 (2.11, 2.11)	264	I11,N19	1.96 (1.96, 1.96)
171	I31,N18	2.3 (2.29, 2.3)	218	G45,I65	2.11 (2.11, 2.11)	265	G45,R93	1.14 (1.14, 1.14)
172	I42,M10	2.29 (2.28, 2.3)	219	G47,J32	2.11 (2.1, 2.12)	266	K27,N19	1.96 (1.95, 1.97)
173	I48,I50	2.28 (2.28, 2.28)	220	F41,J32	2.11 (2.1, 2.12)	267	G47,I70	1.96 (1.95, 1.96)
174	G45,G47	2.28 (2.28, 2.28)	221	E77,I27	2.11 (2.11, 2.11)	268	D64,N39	1.95 (1.95, 1.95)
175	I42,I49	2.28 (2.27, 2.28)	222	G47,M17	2.11 (2.1, 2.11)	269	J18,K72	1.95 (1.95, 1.95)
176	E11,R93	1.26 (1.26, 1.26)	223	I31,J84	2.1 (2.08, 2.12)	270	I45,I51	1.95 (1.95, 1.95)
177	I65,I69	2.26 (2.26, 2.26)	224	J32,M48	2.1 (2.09, 2.1)	271	M50,M81	1.95 (1.95, 1.95)
178	E04,M51	2.26 (2.26, 2.26)	225	M47,M81	2.09 (2.09, 2.09)	272	E79,I38	1.95 (1.94, 1.95)
179	D69,I31	2.26 (2.25, 2.27)	226	D69,E79	2.08 (2.08, 2.09)	273	N18,N28	1.95 (1.95, 1.95)
180	G47,M47	2.26 (2.25, 2.26)	227	N18,N39	2.07 (2.07, 2.07)	274	E79,I31	1.95 (1.94, 1.95)
181	E77,N39	2.25 (2.25, 2.25)	228	E79,R93	1.77 (1.76, 1.77)	275	M06,M51	1.94 (1.94, 1.95)
182	E04,I65	2.25 (2.24, 2.26)	229	J43,J45	2.07 (2.07, 2.08)	276	I38,J96	1.94 (1.94, 1.95)
183	I31,J18	2.25 (2.24, 2.25)	230	F41,R93	1.42 (1.41, 1.44)	277	J44,J84	1.94 (1.94, 1.94)
184	E14,N18	2.23 (2.23, 2.23)	231	J18,J45	2.07 (2.07, 2.07)	278	K82,N40	1.94 (1.94, 1.94)
185	D64,M10	2.23 (2.23, 2.23)	232	I63,J32	2.06 (2.06, 2.06)	279	I44,N19	1.94 (1.93, 1.95)
186	E04,N28	2.23 (2.23, 2.23)	233	I27,I38	2.06 (2.06, 2.06)	280	K59,N40	1.93 (1.93, 1.93)
187	I38,J84	2.23 (2.21, 2.24)	234	D64,K59	2.06 (2.06, 2.06)	281	I47,I51	1.93 (1.93, 1.93)
188	G45,M47	2.22 (2.22, 2.22)	235	I49,I51	2.06 (2.06, 2.06)	282	D64,K72	1.93 (1.93, 1.93)

TABLE S2. Data in the form of comorbidity network in IHD patients (continued)

ID	Disease pair	OER (99% CI)	ID	Disease pair	OER (99% CI)	ID	Disease pair	OER (99% CI)
283	D50,I38	1.93 (1.92, 1.94)	330	I51,N18	1.86 (1.86, 1.86)	377	G31,I67	1.77 (1.77, 1.77)
284	I11,I65	1.93 (1.93, 1.93)	331	G31,G45	1.85 (1.85, 1.86)	378	I44,R93	1.58 (1.56, 1.59)
285	K59,N18	1.93 (1.92, 1.93)	332	H81,R93	1.21 (1.19, 1.22)	379	K59,N28	1.77 (1.76, 1.77)
286	E79,I44	1.92 (1.92, 1.93)	333	G31,H81	1.85 (1.85, 1.85)	380	F41,M48	1.76 (1.76, 1.77)
287	K31,K52	1.92 (1.92, 1.93)	334	I31,J47	1.85 (1.84, 1.86)	381	I70,K76	1.76 (1.76, 1.76)
288	D64,E03	1.92 (1.92, 1.93)	335	J96,K59	1.85 (1.84, 1.85)	382	I45,R93	1.78 (1.76, 1.8)
289	I11,I44	1.92 (1.92, 1.92)	336	H81,M89	1.85 (1.84, 1.85)	383	D69,K59	1.76 (1.75, 1.77)
290	G47,R93	1.76 (1.75, 1.77)	337	I10,R93	1.05 (1.05, 1.05)	384	N28,N39	1.76 (1.76, 1.76)
291	I11,I70	1.92 (1.92, 1.92)	338	D50,I51	1.84 (1.84, 1.84)	385	K59,M89	1.76 (1.75, 1.77)
292	H25,R93	1.63 (1.63, 1.64)	339	E79,I51	1.84 (1.84, 1.84)	386	J18,K59	1.76 (1.75, 1.76)
293	E77,I38	1.92 (1.91, 1.92)	340	E04,I11	1.84 (1.84, 1.84)	387	E78,I65	1.75 (1.75, 1.75)
294	I38,I65	1.92 (1.91, 1.92)	341	D64,E79	1.83 (1.83, 1.83)	388	D69,I42	1.75 (1.74, 1.77)
295	H81,M48	1.91 (1.91, 1.92)	342	N19,N28	1.83 (1.83, 1.84)	389	K59,M48	1.75 (1.75, 1.75)
296	D69,E03	1.91 (1.9, 1.92)	343	I38,K59	1.83 (1.83, 1.84)	390	I45,I48	1.75 (1.75, 1.75)
297	M06,M10	1.91 (1.9, 1.92)	344	J32,J45	1.83 (1.82, 1.85)	391	I38,I50	1.75 (1.75, 1.75)
298	J43,J44	1.91 (1.91, 1.91)	345	I11,R93	1.6 (1.6, 1.6)	392	E79,I45	1.75 (1.75, 1.76)
299	E04,E78	1.91 (1.91, 1.91)	346	G31,M47	1.83 (1.83, 1.83)	393	K29,K31	1.75 (1.75, 1.75)
300	G47,M48	1.91 (1.9, 1.91)	347	E03,E79	1.82 (1.82, 1.83)	394	D69,J84	1.75 (1.74, 1.76)
301	E03,N19	1.9 (1.89, 1.91)	348	I38,I47	1.82 (1.81, 1.83)	395	I65,I67	1.75 (1.75, 1.75)
302	I63,M50	1.9 (1.9, 1.9)	349	E77,J43	1.82 (1.82, 1.82)	396	E78,M50	1.75 (1.75, 1.75)
303	K76,K80	1.9 (1.9, 1.9)	350	I38,J18	1.82 (1.82, 1.82)	397	I38,I70	1.74 (1.74, 1.75)
304	G47,M81	1.9 (1.9, 1.9)	351	I27,R93	1.16 (1.15, 1.16)	398	H26,J32	1.74 (1.73, 1.76)
305	I38,I45	1.9 (1.88, 1.91)	352	I44,K59	1.81 (1.81, 1.82)	399	J84,N28	1.74 (1.74, 1.75)
306	J18,N19	1.9 (1.89, 1.9)	353	I31,R93	2.16 (2.15, 2.18)	400	G47,K76	1.74 (1.74, 1.74)
307	K27,M10	1.89 (1.88, 1.9)	354	M06,M89	1.81 (1.8, 1.82)	401	G31,J43	1.74 (1.74, 1.74)
308	J18,J43	1.89 (1.89, 1.89)	355	I11,K59	1.81 (1.81, 1.81)	402	G47,K31	1.74 (1.74, 1.75)
309	M10,N40	1.89 (1.89, 1.89)	356	K59,K72	1.81 (1.8, 1.81)	403	K25,M89	1.74 (1.73, 1.75)
310	M47,M89	1.89 (1.88, 1.89)	357	E79,N28	1.81 (1.81, 1.81)	404	K31,K72	1.74 (1.74, 1.75)
311	G44,K25	1.88 (1.87, 1.9)	358	J32,K82	1.81 (1.79, 1.82)	405	I65,M48	1.74 (1.74, 1.74)
312	I70,N28	1.88 (1.88, 1.88)	359	D50,D69	1.81 (1.79, 1.82)	406	J84,N40	1.74 (1.74, 1.74)
313	E04,I67	1.88 (1.88, 1.88)	360	J84,K59	1.8 (1.79, 1.81)	407	I44,N18	1.74 (1.73, 1.74)
314	F41,I67	1.88 (1.88, 1.88)	361	D69,I45	1.8 (1.79, 1.81)	408	K27,K59	1.74 (1.73, 1.75)
315	G45,J32	1.88 (1.88, 1.88)	362	I38,N28	1.8 (1.8, 1.8)	409	G44,M89	1.74 (1.73, 1.75)
316	E78,F41	1.88 (1.88, 1.88)	363	J45,J84	1.8 (1.78, 1.82)	410	I70,K59	1.73 (1.73, 1.74)
317	I11,I69	1.88 (1.88, 1.88)	364	I27,K72	1.8 (1.8, 1.8)	411	K21,K29	1.73 (1.73, 1.73)
318	E03,E78	1.87 (1.87, 1.87)	365	I38,R93	1.83 (1.82, 1.84)	412	F41,M89	1.73 (1.72, 1.74)
319	G47,I65	1.87 (1.86, 1.87)	366	F41,I65	1.8 (1.79, 1.8)	413	G47,H26	1.73 (1.72, 1.74)
320	K80,N28	1.87 (1.87, 1.87)	367	K72,N18	1.8 (1.79, 1.8)	414	I50,J96	1.73 (1.73, 1.73)
321	M10,N28	1.87 (1.87, 1.87)	368	K27,M89	1.79 (1.78, 1.8)	415	K59,N19	1.73 (1.72, 1.74)
322	E79,K76	1.87 (1.87, 1.87)	369	H81,I67	1.79 (1.79, 1.79)	416	I69,N39	1.73 (1.73, 1.73)
323	H26,R93	1.28 (1.26, 1.29)	370	E04,E79	1.79 (1.78, 1.79)	417	K25,N19	1.73 (1.72, 1.74)
324	I31,I50	1.87 (1.87, 1.87)	371	I44,M10	1.78 (1.78, 1.79)	418	D69,I48	1.73 (1.73, 1.73)
325	H26,M81	1.87 (1.86, 1.87)	372	I42,J18	1.78 (1.78, 1.78)	419	G45,H81	1.73 (1.72, 1.73)
326	E14,H25	1.87 (1.86, 1.87)	373	I42,R93	1.31 (1.29, 1.33)	420	K76,N40	1.72 (1.72, 1.72)
327	E78,K76	1.86 (1.86, 1.86)	374	E79,G47	1.77 (1.77, 1.78)	421	I47,R93	1.17 (1.16, 1.19)
328	F41,M81	1.86 (1.86, 1.87)	375	E79,I70	1.77 (1.77, 1.77)	422	G47,M51	1.72 (1.72, 1.72)
329	K52,N19	1.86 (1.85, 1.86)	376	J43,N40	1.77 (1.77, 1.77)	423	E03,I42	1.72 (1.71, 1.74)

TABLE S2. Data in the form of comorbidity network in IHD patients (continued)

ID	Disease pair	OER (99% CI)	ID	Disease pair	OER (99% CI)	ID	Disease pair	OER (99% CI)
424	E03,K59	1.72 (1.71, 1.73)	471	I38,J43	1.66 (1.66, 1.67)	518	H26,I11	1.62 (1.62, 1.62)
425	I31,K59	1.72 (1.71, 1.73)	472	I44,N40	1.66 (1.66, 1.67)	519	I11,I48	1.62 (1.62, 1.62)
426	G47,M89	1.72 (1.71, 1.72)	473	I65,N19	1.66 (1.66, 1.67)	520	E77,K31	1.62 (1.62, 1.62)
427	K52,N39	1.72 (1.71, 1.72)	474	I48,K72	1.66 (1.66, 1.66)	521	G47,I47	1.62 (1.61, 1.63)
428	G47,I49	1.71 (1.71, 1.71)	475	D64,K31	1.66 (1.66, 1.66)	522	E04,I38	1.62 (1.61, 1.63)
429	I65,I70	1.71 (1.71, 1.71)	476	G45,M48	1.66 (1.66, 1.66)	523	I38,J47	1.62 (1.61, 1.62)
430	E79,I48	1.71 (1.71, 1.71)	477	K27,K72	1.66 (1.65, 1.67)	524	E79,I65	1.62 (1.62, 1.62)
431	J43,N28	1.71 (1.71, 1.71)	478	J84,K72	1.66 (1.65, 1.67)	525	K59,M17	1.62 (1.61, 1.62)
432	E79,I47	1.71 (1.7, 1.71)	479	I49,R93	1.39 (1.39, 1.39)	526	K27,K52	1.62 (1.61, 1.62)
433	D69,I38	1.7 (1.7, 1.71)	480	I27,I50	1.66 (1.66, 1.66)	527	D50,K59	1.62 (1.61, 1.63)
434	I38,K27	1.7 (1.69, 1.71)	481	I31,N28	1.66 (1.65, 1.66)	528	I65,J32	1.62 (1.61, 1.62)
435	K72,M10	1.7 (1.69, 1.7)	482	J32,K76	1.65 (1.65, 1.66)	529	E78,M47	1.62 (1.62, 1.62)
436	G45,I70	1.7 (1.7, 1.7)	483	F41,I69	1.65 (1.65, 1.66)	530	I45,K59	1.62 (1.6, 1.63)
437	I27,J18	1.7 (1.7, 1.7)	484	I11,I38	1.65 (1.65, 1.66)	531	E78,I70	1.61 (1.61, 1.61)
438	I65,M50	1.7 (1.69, 1.7)	485	I69,N19	1.65 (1.65, 1.66)	532	I45,N28	1.61 (1.61, 1.62)
439	E77,N28	1.69 (1.69, 1.7)	486	I10,I11	1.65 (1.65, 1.65)	533	I67,R93	1.29 (1.29, 1.29)
440	I70,K82	1.69 (1.69, 1.7)	487	K76,M50	1.65 (1.65, 1.65)	534	I70,N40	1.61 (1.61, 1.61)
441	D64,I38	1.69 (1.69, 1.7)	488	I11,N28	1.65 (1.65, 1.65)	535	E14,I11	1.61 (1.61, 1.61)
442	I67,M48	1.69 (1.69, 1.69)	489	D64,J18	1.65 (1.65, 1.65)	536	K76,M17	1.61 (1.61, 1.61)
443	F41,K82	1.69 (1.68, 1.7)	490	I11,I31	1.65 (1.64, 1.65)	537	I70,J32	1.61 (1.61, 1.61)
444	E03,E77	1.69 (1.69, 1.69)	491	K25,K52	1.64 (1.64, 1.65)	538	E04,I49	1.6 (1.6, 1.61)
445	E03,M81	1.69 (1.69, 1.69)	492	M81,N39	1.64 (1.64, 1.64)	539	F41,M17	1.6 (1.59, 1.61)
446	K25,K59	1.69 (1.68, 1.69)	493	G45,I63	1.64 (1.64, 1.64)	540	M06,M50	1.6 (1.59, 1.61)
447	I70,M50	1.69 (1.69, 1.69)	494	I50,R93	1.1 (1.1, 1.1)	541	I48,N19	1.6 (1.6, 1.6)
448	I44,K72	1.69 (1.68, 1.69)	495	E04,I63	1.64 (1.64, 1.64)	542	I69,R93	1.17 (1.16, 1.17)
449	I31,I45	1.69 (1.67, 1.7)	496	G31,I45	1.64 (1.63, 1.64)	543	E04,G44	1.6 (1.59, 1.62)
450	I51,N19	1.68 (1.68, 1.68)	497	G47,J45	1.64 (1.63, 1.65)	544	I70,R93	1.72 (1.72, 1.73)
451	E77,I42	1.68 (1.68, 1.69)	498	I31,I44	1.63 (1.62, 1.65)	545	K59,M51	1.6 (1.6, 1.6)
452	E77,M10	1.68 (1.68, 1.68)	499	I51,R93	1.45 (1.45, 1.45)	546	G31,N28	1.6 (1.6, 1.6)
453	I47,K72	1.68 (1.67, 1.69)	500	J32,K21	1.63 (1.63, 1.64)	547	I49,I50	1.6 (1.6, 1.6)
454	G31,N40	1.68 (1.68, 1.68)	501	I47,I48	1.63 (1.63, 1.63)	548	J84,M81	1.6 (1.59, 1.6)
455	I27,I48	1.68 (1.68, 1.68)	502	I38,N18	1.63 (1.63, 1.64)	549	I50,N18	1.6 (1.6, 1.6)
456	I67,J32	1.68 (1.68, 1.68)	503	I63,M47	1.63 (1.63, 1.63)	550	I45,J84	1.59 (1.57, 1.62)
457	I63,I70	1.68 (1.68, 1.68)	504	I11,I49	1.63 (1.63, 1.63)	551	I45,N40	1.59 (1.59, 1.6)
458	I38,K72	1.68 (1.67, 1.68)	505	G44,I63	1.63 (1.63, 1.63)	552	J84,N19	1.59 (1.58, 1.61)
459	E14,I65	1.67 (1.67, 1.68)	506	F41,M51	1.63 (1.63, 1.63)	553	K76,M89	1.59 (1.59, 1.59)
460	G44,M48	1.67 (1.67, 1.68)	507	I27,I42	1.63 (1.62, 1.63)	554	E79,I49	1.59 (1.59, 1.59)
461	E79,K59	1.67 (1.67, 1.67)	508	I63,R93	1.29 (1.29, 1.29)	555	E14,N39	1.59 (1.59, 1.59)
462	I48,R93	1.16 (1.16, 1.16)	509	E77,K80	1.63 (1.63, 1.63)	556	F41,K29	1.59 (1.59, 1.59)
463	G31,I70	1.67 (1.67, 1.67)	510	J32,N28	1.63 (1.62, 1.63)	557	G47,I63	1.59 (1.59, 1.59)
464	G47,K82	1.67 (1.66, 1.68)	511	E78,M17	1.62 (1.62, 1.62)	558	K27,N39	1.59 (1.58, 1.59)
465	E03,H26	1.67 (1.66, 1.68)	512	G45,M89	1.62 (1.62, 1.63)	559	J84,K21	1.59 (1.58, 1.6)
466	F41,I70	1.67 (1.67, 1.67)	513	I50,N19	1.62 (1.62, 1.62)	560	K31,K82	1.59 (1.58, 1.59)
467	M06,M48	1.67 (1.66, 1.67)	514	E03,M06	1.62 (1.61, 1.64)	561	G31,J42	1.59 (1.59, 1.59)
468	D69,N18	1.67 (1.66, 1.67)	515	I65,R93	1.46 (1.45, 1.47)	562	E77,K52	1.59 (1.59, 1.59)
469	M89,N28	1.67 (1.67, 1.67)	516	I44,I50	1.62 (1.62, 1.62)	563	I44,N28	1.59 (1.58, 1.59)
470	D69,N39	1.67 (1.66, 1.67)	517	J96,K27	1.62 (1.61, 1.63)	564	I31,K82	1.59 (1.57, 1.6)

TABLE S2. Data in the form of comorbidity network in IHD patients (continued)

ID	Disease pair	OER (99% CI)	ID	Disease pair	OER (99% CI)	ID	Disease pair	OER (99% CI)
565	J32,K59	1.58 (1.58, 1.59)	612	J45,K59	1.55 (1.54, 1.56)	659	D64,J96	1.52 (1.51, 1.52)
566	E77,I69	1.58 (1.58, 1.58)	613	K21,M89	1.55 (1.54, 1.56)	660	H26,N19	1.52 (1.5, 1.53)
567	H26,M17	1.58 (1.57, 1.59)	614	I45,I70	1.55 (1.55, 1.55)	661	I67,M89	1.51 (1.51, 1.51)
568	E79,M17	1.58 (1.58, 1.58)	615	D69,N28	1.55 (1.55, 1.55)	662	J96,N40	1.51 (1.51, 1.51)
569	D50,E03	1.58 (1.56, 1.59)	616	E03,I45	1.55 (1.53, 1.56)	663	J18,K27	1.51 (1.51, 1.52)
570	I11,K76	1.58 (1.58, 1.58)	617	J44,N40	1.55 (1.55, 1.55)	664	H81,I63	1.51 (1.51, 1.51)
571	E03,I38	1.58 (1.57, 1.59)	618	D69,J96	1.55 (1.54, 1.55)	665	J42,N40	1.51 (1.51, 1.51)
572	G44,H26	1.58 (1.56, 1.59)	619	I67,K59	1.55 (1.54, 1.55)	666	E04,K80	1.51 (1.51, 1.51)
573	J18,R93	1.6 (1.6, 1.6)	620	J42,M89	1.55 (1.54, 1.55)	667	K72,K76	1.51 (1.51, 1.51)
574	I69,N40	1.58 (1.58, 1.58)	621	E78,I11	1.54 (1.54, 1.54)	668	G44,K21	1.51 (1.5, 1.52)
575	F41,I63	1.58 (1.57, 1.58)	622	G31,I31	1.54 (1.54, 1.55)	669	E04,N18	1.51 (1.5, 1.51)
576	I27,N40	1.57 (1.57, 1.57)	623	E78,M48	1.54 (1.54, 1.54)	670	E11,N39	1.51 (1.51, 1.51)
577	K72,N39	1.57 (1.57, 1.58)	624	M06,N39	1.54 (1.53, 1.55)	671	J47,K21	1.51 (1.5, 1.51)
578	E77,J44	1.57 (1.57, 1.57)	625	H26,K59	1.54 (1.53, 1.55)	672	I47,I70	1.51 (1.51, 1.51)
579	D69,K82	1.57 (1.56, 1.58)	626	I10,I69	1.54 (1.54, 1.54)	673	I70,M81	1.51 (1.5, 1.51)
580	I11,I63	1.57 (1.57, 1.57)	627	J47,K59	1.54 (1.53, 1.54)	674	M89,N39	1.5 (1.5, 1.51)
581	D64,M81	1.57 (1.57, 1.57)	628	E78,J32	1.54 (1.54, 1.54)	675	E04,G45	1.5 (1.5, 1.51)
582	K21,K27	1.57 (1.56, 1.58)	629	J32,R93	2.17 (2.16, 2.19)	676	H26,M89	1.5 (1.49, 1.52)
583	K52,K72	1.57 (1.57, 1.57)	630	D64,I51	1.54 (1.54, 1.54)	677	E77,M81	1.5 (1.5, 1.5)
584	I63,K59	1.57 (1.57, 1.57)	631	G31,M81	1.53 (1.53, 1.54)	678	E14,E77	1.5 (1.5, 1.5)
585	I67,M17	1.57 (1.57, 1.57)	632	E03,N39	1.53 (1.53, 1.54)	679	I27,K59	1.5 (1.5, 1.5)
586	I70,M47	1.57 (1.57, 1.57)	633	K25,M51	1.53 (1.53, 1.53)	680	J47,K72	1.5 (1.5, 1.51)
587	I11,I45	1.57 (1.56, 1.57)	634	G31,I65	1.53 (1.53, 1.53)	681	M89,N40	1.5 (1.5, 1.5)
588	I69,N18	1.57 (1.56, 1.57)	635	E03,M17	1.53 (1.52, 1.54)	682	E11,N19	1.5 (1.5, 1.5)
589	I69,I70	1.56 (1.56, 1.57)	636	G47,K52	1.53 (1.53, 1.54)	683	I45,M10	1.5 (1.49, 1.51)
590	H26,I70	1.56 (1.56, 1.57)	637	E04,K31	1.53 (1.52, 1.54)	684	E77,I48	1.5 (1.5, 1.5)
591	G31,G44	1.56 (1.56, 1.57)	638	N18,N40	1.53 (1.53, 1.53)	685	J32,M51	1.5 (1.49, 1.5)
592	D64,N28	1.56 (1.56, 1.56)	639	K76,M48	1.53 (1.53, 1.53)	686	I45,K72	1.5 (1.49, 1.51)
593	I70,M48	1.56 (1.56, 1.56)	640	I31,K27	1.53 (1.51, 1.55)	687	D50,E79	1.5 (1.49, 1.5)
594	I45,K82	1.56 (1.55, 1.58)	641	E03,M50	1.53 (1.52, 1.54)	688	G31,J84	1.49 (1.49, 1.5)
595	K25,M48	1.56 (1.56, 1.56)	642	G47,I44	1.53 (1.52, 1.54)	689	K52,M89	1.49 (1.49, 1.5)
596	H81,K31	1.56 (1.56, 1.57)	643	D69,J32	1.53 (1.52, 1.54)	690	M48,N28	1.49 (1.49, 1.49)
597	E03,I70	1.56 (1.56, 1.56)	644	H25,M81	1.53 (1.52, 1.53)	691	K25,K29	1.49 (1.49, 1.49)
598	D64,E14	1.56 (1.56, 1.56)	645	I47,I50	1.53 (1.53, 1.53)	692	K76,M10	1.49 (1.49, 1.49)
599	N19,N40	1.56 (1.56, 1.56)	646	E11,E78	1.52 (1.52, 1.52)	693	K52,M06	1.49 (1.48, 1.5)
600	I49,K59	1.56 (1.56, 1.56)	647	J42,R93	1.13 (1.13, 1.13)	694	E04,N39	1.49 (1.48, 1.5)
601	I65,K76	1.56 (1.56, 1.56)	648	E78,G45	1.52 (1.52, 1.52)	695	J32,J84	1.49 (1.47, 1.51)
602	D69,E04	1.56 (1.55, 1.57)	649	M06,M47	1.52 (1.52, 1.53)	696	G44,I65	1.49 (1.48, 1.5)
603	E03,K72	1.56 (1.55, 1.56)	650	I65,N40	1.52 (1.52, 1.52)	697	K82,M89	1.49 (1.48, 1.5)
604	I51,M10	1.55 (1.55, 1.56)	651	K21,M47	1.52 (1.52, 1.52)	698	J43,K59	1.49 (1.49, 1.49)
605	I44,I65	1.55 (1.55, 1.56)	652	D64,K52	1.52 (1.52, 1.52)	699	K21,K72	1.49 (1.48, 1.49)
606	I65,M47	1.55 (1.55, 1.55)	653	I38,J45	1.52 (1.51, 1.53)	700	E79,N40	1.49 (1.49, 1.49)
607	H25,J32	1.55 (1.55, 1.56)	654	J47,N28	1.52 (1.52, 1.52)	701	E14,K52	1.49 (1.48, 1.49)
608	K29,K59	1.55 (1.55, 1.55)	655	K82,M50	1.52 (1.51, 1.53)	702	I49,I70	1.49 (1.49, 1.49)
609	I44,I70	1.55 (1.55, 1.55)	656	F41,N39	1.52 (1.51, 1.52)	703	E77,N40	1.49 (1.49, 1.49)
610	I70,M17	1.55 (1.55, 1.55)	657	E79,J96	1.52 (1.52, 1.52)	704	G47,I69	1.49 (1.48, 1.49)
611	E03,J84	1.55 (1.53, 1.57)	658	G47,N28	1.52 (1.51, 1.52)	705	K31,M89	1.49 (1.48, 1.49)

TABLE S2. Data in the form of comorbidity network in IHD patients (continued)

ID	Disease pair	OER (99% CI)	ID	Disease pair	OER (99% CI)	ID	Disease pair	OER (99% CI)
706	E77,G31	1.48 (1.48, 1.49)	753	D50,N28	1.46 (1.45, 1.46)	800	K59,K82	1.43 (1.42, 1.44)
707	K21,K82	1.48 (1.48, 1.49)	754	G31,I38	1.46 (1.45, 1.46)	801	E04,M89	1.43 (1.42, 1.44)
708	I65,K59	1.48 (1.48, 1.49)	755	G47,K29	1.46 (1.46, 1.46)	802	I45,J43	1.43 (1.43, 1.44)
709	E78,I67	1.48 (1.48, 1.48)	756	I70,J84	1.45 (1.45, 1.46)	803	I51,J96	1.43 (1.43, 1.43)
710	E78,H26	1.48 (1.48, 1.48)	757	I42,K27	1.45 (1.43, 1.47)	804	E79,I27	1.43 (1.43, 1.43)
711	J43,M89	1.48 (1.48, 1.49)	758	I38,M48	1.45 (1.45, 1.46)	805	I44,I48	1.43 (1.43, 1.43)
712	K21,M48	1.48 (1.48, 1.48)	759	I48,I49	1.45 (1.45, 1.45)	806	M17,N39	1.43 (1.42, 1.43)
713	I45,I50	1.48 (1.48, 1.48)	760	K80,K82	1.45 (1.45, 1.45)	807	I11,K82	1.43 (1.42, 1.43)
714	I11,I50	1.48 (1.48, 1.48)	761	K82,M47	1.45 (1.45, 1.45)	808	E79,M48	1.43 (1.43, 1.43)
715	J43,R93	1.82 (1.81, 1.82)	762	I11,I67	1.45 (1.45, 1.45)	809	G31,H26	1.43 (1.42, 1.43)
716	F41,K52	1.48 (1.47, 1.49)	763	D69,I51	1.45 (1.45, 1.45)	810	F41,I47	1.43 (1.42, 1.44)
717	G47,N39	1.48 (1.48, 1.48)	764	D64,J84	1.45 (1.45, 1.45)	811	E78,K82	1.43 (1.42, 1.43)
718	D69,I44	1.48 (1.47, 1.49)	765	G44,K29	1.45 (1.45, 1.45)	812	K59,M50	1.43 (1.42, 1.43)
719	J47,N40	1.48 (1.48, 1.48)	766	J47,R93	1.62 (1.61, 1.63)	813	G44,M06	1.42 (1.41, 1.44)
720	E04,I45	1.48 (1.46, 1.49)	767	K72,K82	1.45 (1.44, 1.46)	814	K21,K76	1.42 (1.42, 1.43)
721	I27,N19	1.48 (1.47, 1.48)	768	I31,J44	1.45 (1.45, 1.45)	815	E04,I47	1.42 (1.41, 1.44)
722	E14,E78	1.48 (1.48, 1.48)	769	D69,G31	1.45 (1.44, 1.45)	816	K29,M06	1.42 (1.42, 1.42)
723	J42,K59	1.48 (1.47, 1.48)	770	H26,N39	1.45 (1.44, 1.45)	817	J96,R93	1.44 (1.43, 1.45)
724	J47,K27	1.48 (1.46, 1.49)	771	E78,M51	1.45 (1.45, 1.45)	818	E04,G31	1.42 (1.42, 1.43)
725	J47,K82	1.48 (1.47, 1.48)	772	D69,K52	1.45 (1.44, 1.45)	819	I65,M10	1.42 (1.42, 1.43)
726	J32,J43	1.47 (1.47, 1.48)	773	D69,H26	1.45 (1.43, 1.46)	820	E04,J84	1.42 (1.4, 1.44)
727	E14,I69	1.47 (1.47, 1.48)	774	G31,M51	1.45 (1.45, 1.45)	821	E03,H25	1.42 (1.41, 1.43)
728	E77,I51	1.47 (1.47, 1.47)	775	I47,N19	1.45 (1.44, 1.46)	822	J43,K27	1.42 (1.42, 1.43)
729	G44,K31	1.47 (1.46, 1.48)	776	I51,I70	1.45 (1.45, 1.45)	823	J84,K76	1.42 (1.42, 1.42)
730	E11,I11	1.47 (1.47, 1.47)	777	I67,I70	1.45 (1.45, 1.45)	824	I31,I49	1.42 (1.42, 1.42)
731	J44,R93	1.18 (1.18, 1.18)	778	J47,M06	1.44 (1.44, 1.45)	825	I38,K52	1.42 (1.41, 1.42)
732	H25,M17	1.47 (1.46, 1.47)	779	K21,M50	1.44 (1.44, 1.45)	826	E04,I44	1.42 (1.41, 1.43)
733	J45,K76	1.47 (1.46, 1.47)	780	I27,K27	1.44 (1.44, 1.45)	827	H81,K52	1.42 (1.41, 1.42)
734	K76,M47	1.47 (1.46, 1.47)	781	I67,M51	1.44 (1.44, 1.44)	828	D69,J43	1.42 (1.41, 1.42)
735	E03,I11	1.46 (1.46, 1.47)	782	E04,H81	1.44 (1.43, 1.45)	829	G31,I44	1.42 (1.41, 1.42)
736	I49,I65	1.46 (1.46, 1.47)	783	J84,R93	1.84 (1.82, 1.87)	830	I49,N19	1.42 (1.42, 1.42)
737	I11,N40	1.46 (1.46, 1.46)	784	I63,I67	1.44 (1.44, 1.44)	831	E03,I44	1.42 (1.41, 1.43)
738	I69,J32	1.46 (1.46, 1.47)	785	K31,M06	1.44 (1.43, 1.45)	832	E79,J84	1.42 (1.41, 1.42)
739	K27,N28	1.46 (1.46, 1.46)	786	E78,H81	1.44 (1.44, 1.44)	833	E79,N39	1.42 (1.42, 1.42)
740	J32,N40	1.46 (1.46, 1.46)	787	F41,K76	1.44 (1.44, 1.44)	834	E11,I69	1.42 (1.42, 1.42)
741	K25,N28	1.46 (1.46, 1.46)	788	I51,J84	1.44 (1.44, 1.44)	835	I38,M10	1.42 (1.41, 1.42)
742	E78,G44	1.46 (1.46, 1.46)	789	G31,I11	1.44 (1.44, 1.44)	836	E79,I50	1.42 (1.41, 1.42)
743	J45,R93	1.26 (1.25, 1.28)	790	K52,N18	1.44 (1.44, 1.44)	837	K29,M89	1.41 (1.41, 1.42)
744	D69,M10	1.46 (1.45, 1.47)	791	I48,I69	1.44 (1.44, 1.44)	838	F41,H26	1.41 (1.4, 1.43)
745	I51,K72	1.46 (1.46, 1.46)	792	E78,K21	1.44 (1.44, 1.44)	839	K25,K82	1.41 (1.4, 1.42)
746	E14,K80	1.46 (1.46, 1.46)	793	I38,J44	1.44 (1.44, 1.44)	840	D69,N40	1.41 (1.41, 1.41)
747	I65,N28	1.46 (1.46, 1.46)	794	K59,K80	1.44 (1.43, 1.44)	841	D69,G47	1.41 (1.41, 1.42)
748	G45,I69	1.46 (1.46, 1.46)	795	I65,K82	1.44 (1.43, 1.44)	842	K80,N19	1.41 (1.41, 1.41)
749	I63,M48	1.46 (1.46, 1.46)	796	J32,K31	1.43 (1.43, 1.44)	843	E11,I65	1.41 (1.41, 1.41)
750	G47,I45	1.46 (1.45, 1.47)	797	I70,N18	1.43 (1.43, 1.43)	844	I38,N40	1.41 (1.41, 1.41)
751	F41,J45	1.46 (1.45, 1.47)	798	I27,I51	1.43 (1.43, 1.43)	845	I11,M17	1.41 (1.41, 1.41)
752	K59,M47	1.46 (1.45, 1.46)	799	K82,M48	1.43 (1.43, 1.44)	846	J18,N18	1.41 (1.41, 1.41)

TABLE S2. Data in the form of comorbidity network in IHD patients (continued)

ID	Disease pair	OER (99% CI)	ID	Disease pair	OER (99% CI)	ID	Disease pair	OER (99% CI)
847	D50,N39	1.41 (1.4, 1.42)	894	K31,N28	1.38 (1.38, 1.38)	941	K31,K76	1.36 (1.36, 1.36)
848	J18,N28	1.41 (1.41, 1.41)	895	K21,M81	1.38 (1.38, 1.39)	942	K52,K76	1.36 (1.36, 1.36)
849	I11,K80	1.41 (1.41, 1.41)	896	I45,M50	1.38 (1.37, 1.39)	943	E03,I65	1.36 (1.36, 1.37)
850	E14,M10	1.41 (1.4, 1.41)	897	I67,K21	1.38 (1.38, 1.38)	944	H26,M10	1.36 (1.35, 1.37)
851	G45,K21	1.41 (1.41, 1.41)	898	D50,K72	1.38 (1.37, 1.39)	945	I38,K25	1.36 (1.35, 1.37)
852	K21,R93	1.64 (1.63, 1.65)	899	I10,M10	1.38 (1.38, 1.38)	946	I70,K80	1.36 (1.36, 1.36)
853	K27,N18	1.41 (1.4, 1.41)	900	I45,M48	1.38 (1.38, 1.39)	947	E77,I44	1.36 (1.36, 1.36)
854	K25,M81	1.41 (1.4, 1.41)	901	J96,N28	1.38 (1.38, 1.38)	948	E14,I10	1.36 (1.36, 1.36)
855	I51,N28	1.4 (1.4, 1.41)	902	E79,I69	1.38 (1.38, 1.38)	949	E03,M47	1.36 (1.36, 1.36)
856	J96,N18	1.4 (1.4, 1.41)	903	J32,J47	1.38 (1.37, 1.39)	950	I45,N18	1.36 (1.35, 1.36)
857	E79,K82	1.4 (1.4, 1.41)	904	K25,K72	1.38 (1.37, 1.39)	951	I49,J84	1.36 (1.35, 1.36)
858	E04,E11	1.4 (1.4, 1.4)	905	I70,N19	1.38 (1.38, 1.38)	952	J84,M48	1.36 (1.35, 1.36)
859	J42,M51	1.4 (1.4, 1.4)	906	J84,K82	1.38 (1.36, 1.4)	953	I69,J18	1.36 (1.36, 1.36)
860	G45,M51	1.4 (1.4, 1.4)	907	I44,I69	1.38 (1.38, 1.38)	954	D69,I47	1.36 (1.35, 1.37)
861	J42,M47	1.4 (1.4, 1.4)	908	K72,N28	1.38 (1.38, 1.38)	955	H81,I65	1.36 (1.35, 1.36)
862	I11,I47	1.4 (1.4, 1.4)	909	M06,N19	1.38 (1.37, 1.39)	956	E14,M89	1.36 (1.35, 1.36)
863	J84,N18	1.4 (1.39, 1.41)	910	K27,R93	2.31 (2.29, 2.33)	957	E79,J32	1.36 (1.35, 1.36)
864	I11,K21	1.4 (1.4, 1.4)	911	I70,M89	1.38 (1.38, 1.38)	958	E03,J32	1.36 (1.34, 1.37)
865	J32,J42	1.4 (1.4, 1.4)	912	K27,K82	1.38 (1.36, 1.39)	959	K59,M10	1.36 (1.35, 1.36)
866	K52,M48	1.4 (1.4, 1.4)	913	E79,K80	1.38 (1.38, 1.38)	960	H25,M06	1.36 (1.35, 1.36)
867	I38,M81	1.4 (1.39, 1.4)	914	H25,I70	1.38 (1.38, 1.38)	961	D69,J18	1.36 (1.35, 1.36)
868	E03,I47	1.39 (1.38, 1.41)	915	F41,I49	1.38 (1.38, 1.38)	962	D69,K27	1.35 (1.34, 1.37)
869	I38,K82	1.39 (1.38, 1.4)	916	G31,J18	1.38 (1.38, 1.38)	963	I10,N18	1.35 (1.35, 1.35)
870	D50,K21	1.39 (1.38, 1.4)	917	M48,N39	1.38 (1.37, 1.38)	964	E03,M10	1.35 (1.35, 1.36)
871	H26,I63	1.39 (1.39, 1.39)	918	J18,J44	1.37 (1.37, 1.37)	965	E03,I51	1.35 (1.35, 1.36)
872	I48,M10	1.39 (1.39, 1.39)	919	K76,M51	1.37 (1.37, 1.37)	966	I70,K21	1.35 (1.35, 1.35)
873	F41,K25	1.39 (1.38, 1.4)	920	K25,M50	1.37 (1.37, 1.38)	967	D50,J18	1.35 (1.35, 1.36)
874	K25,M17	1.39 (1.38, 1.4)	921	E03,K76	1.37 (1.37, 1.37)	968	G31,H25	1.35 (1.35, 1.35)
875	D69,I70	1.39 (1.39, 1.39)	922	D69,M89	1.37 (1.36, 1.38)	969	E78,M81	1.35 (1.35, 1.35)
876	J32,M17	1.39 (1.38, 1.4)	923	J42,M48	1.37 (1.37, 1.37)	970	J43,K72	1.35 (1.35, 1.35)
877	G47,K25	1.39 (1.38, 1.4)	924	G31,K76	1.37 (1.37, 1.37)	971	K29,R93	1.15 (1.15, 1.16)
878	E79,K52	1.39 (1.39, 1.39)	925	E11,K76	1.37 (1.37, 1.37)	972	I45,I65	1.35 (1.34, 1.36)
879	K25,R93	1.85 (1.84, 1.87)	926	G31,N39	1.37 (1.37, 1.37)	973	I45,M89	1.35 (1.34, 1.36)
880	D69,I49	1.39 (1.39, 1.39)	927	H26,M47	1.37 (1.37, 1.37)	974	E79,H26	1.35 (1.34, 1.35)
881	E78,K59	1.39 (1.39, 1.39)	928	H26,I65	1.37 (1.36, 1.38)	975	E79,J45	1.35 (1.34, 1.35)
882	E03,I49	1.39 (1.39, 1.39)	929	D69,H25	1.37 (1.36, 1.37)	976	K31,R93	1.98 (1.97, 1.99)
883	I31,K80	1.39 (1.39, 1.39)	930	M17,N28	1.37 (1.37, 1.37)	977	G44,I70	1.35 (1.35, 1.35)
884	G31,M17	1.39 (1.39, 1.39)	931	J44,K59	1.37 (1.37, 1.37)	978	I67,K82	1.35 (1.35, 1.35)
885	I70,M10	1.39 (1.39, 1.39)	932	D69,K76	1.37 (1.37, 1.37)	979	E11,M10	1.35 (1.35, 1.35)
886	E11,I70	1.39 (1.39, 1.39)	933	K25,M47	1.37 (1.36, 1.37)	980	K29,M47	1.35 (1.35, 1.35)
887	G44,K52	1.39 (1.38, 1.39)	934	I67,K76	1.37 (1.37, 1.37)	981	D50,M81	1.35 (1.34, 1.35)
888	G45,I67	1.39 (1.39, 1.39)	935	E77,I70	1.37 (1.37, 1.37)	982	E11,I10	1.35 (1.35, 1.35)
889	K59,K76	1.39 (1.38, 1.39)	936	D64,K80	1.37 (1.36, 1.37)	983	E79,J18	1.35 (1.34, 1.35)
890	I50,K72	1.39 (1.39, 1.39)	937	K52,K82	1.37 (1.36, 1.37)	984	I44,J84	1.35 (1.33, 1.36)
891	G44,K82	1.38 (1.37, 1.4)	938	D69,K80	1.36 (1.36, 1.37)	985	G47,H25	1.34 (1.34, 1.35)
892	D64,I11	1.38 (1.38, 1.39)	939	H26,M50	1.36 (1.36, 1.37)	986	H26,K76	1.34 (1.34, 1.35)
893	E77,I50	1.38 (1.38, 1.38)	940	I45,K76	1.36 (1.36, 1.37)	987	J45,K82	1.34 (1.33, 1.36)

TABLE S2. Data in the form of comorbidity network in IHD patients (continued)

ID	Disease pair	OER (99% CI)	ID	Disease pair	OER (99% CI)	ID	Disease pair	OER (99% CI)
988	E03,M48	1.34 (1.34, 1.35)	1035	H26,M48	1.32 (1.32, 1.33)	1082	K52,R93	1.61 (1.6, 1.62)
989	H26,N28	1.34 (1.34, 1.34)	1036	E04,M06	1.32 (1.31, 1.34)	1083	D64,H26	1.3 (1.3, 1.31)
990	H25,K59	1.34 (1.34, 1.35)	1037	D50,I50	1.32 (1.32, 1.32)	1084	E79,G31	1.3 (1.3, 1.31)
991	E79,M89	1.34 (1.34, 1.34)	1038	J42,M17	1.32 (1.32, 1.32)	1085	K80,N39	1.3 (1.3, 1.31)
992	H25,I65	1.34 (1.34, 1.34)	1039	I65,N18	1.32 (1.32, 1.32)	1086	I67,N40	1.3 (1.3, 1.3)
993	M50,N28	1.34 (1.34, 1.34)	1040	I38,J32	1.32 (1.31, 1.33)	1087	I67,K29	1.3 (1.3, 1.3)
994	E78,M10	1.34 (1.34, 1.34)	1041	M81,N28	1.32 (1.32, 1.32)	1088	I38,K31	1.3 (1.3, 1.31)
995	E04,K52	1.34 (1.33, 1.35)	1042	E79,K27	1.32 (1.32, 1.33)	1089	J47,K31	1.3 (1.3, 1.31)
996	E04,J47	1.34 (1.33, 1.35)	1043	K52,M50	1.32 (1.32, 1.32)	1090	E78,J45	1.3 (1.3, 1.3)
997	G44,I69	1.34 (1.33, 1.34)	1044	J43,K25	1.32 (1.32, 1.32)	1091	D50,K82	1.3 (1.29, 1.31)
998	I51,J18	1.34 (1.34, 1.34)	1045	D69,M81	1.32 (1.32, 1.32)	1092	H26,N40	1.3 (1.3, 1.3)
999	I65,J84	1.34 (1.33, 1.35)	1046	D64,G31	1.32 (1.32, 1.32)	1093	F41,G31	1.3 (1.3, 1.3)
1000	H26,K82	1.34 (1.32, 1.35)	1047	G44,M17	1.32 (1.31, 1.33)	1094	G31,G47	1.3 (1.3, 1.3)
1001	K52,M47	1.34 (1.34, 1.34)	1048	I10,I65	1.32 (1.32, 1.32)	1095	K21,M17	1.3 (1.29, 1.31)
1002	D50,K52	1.34 (1.33, 1.34)	1049	J42,K21	1.32 (1.32, 1.32)	1096	K52,M51	1.3 (1.3, 1.3)
1003	I69,M10	1.33 (1.33, 1.34)	1050	D64,I50	1.32 (1.32, 1.32)	1097	I47,J96	1.3 (1.3, 1.3)
1004	E78,M89	1.33 (1.33, 1.33)	1051	K80,N18	1.32 (1.32, 1.32)	1098	K25,N18	1.3 (1.3, 1.3)
1005	J43,M48	1.33 (1.33, 1.33)	1052	E04,J18	1.32 (1.31, 1.32)	1099	I69,M81	1.3 (1.3, 1.3)
1006	D69,M50	1.33 (1.33, 1.34)	1053	I44,K80	1.32 (1.31, 1.32)	1100	G45,H26	1.3 (1.3, 1.3)
1007	K72,M89	1.33 (1.33, 1.34)	1054	K52,M10	1.32 (1.31, 1.32)	1101	G31,K27	1.3 (1.29, 1.3)
1008	I11,J32	1.33 (1.33, 1.34)	1055	H26,M51	1.32 (1.31, 1.32)	1102	I67,K31	1.3 (1.3, 1.3)
1009	I69,M50	1.33 (1.33, 1.34)	1056	I48,J96	1.31 (1.31, 1.32)	1103	G45,K76	1.3 (1.3, 1.3)
1010	G45,K82	1.33 (1.33, 1.34)	1057	M51,N39	1.31 (1.31, 1.32)	1104	E14,M81	1.3 (1.3, 1.3)
1011	H81,I70	1.33 (1.33, 1.33)	1058	K21,N39	1.31 (1.31, 1.32)	1105	I45,K80	1.3 (1.29, 1.3)
1012	K31,N39	1.33 (1.33, 1.34)	1059	D50,I42	1.31 (1.29, 1.33)	1106	D69,M51	1.3 (1.29, 1.3)
1013	D64,I42	1.33 (1.33, 1.34)	1060	K52,N28	1.31 (1.31, 1.31)	1107	K82,M17	1.3 (1.29, 1.3)
1014	E04,E14	1.33 (1.32, 1.34)	1061	J42,K52	1.31 (1.31, 1.31)	1108	G45,M81	1.3 (1.3, 1.3)
1015	K27,M48	1.33 (1.33, 1.34)	1062	E78,I63	1.31 (1.31, 1.31)	1109	E03,K21	1.3 (1.29, 1.3)
1016	D69,M48	1.33 (1.33, 1.33)	1063	D69,K31	1.31 (1.31, 1.32)	1110	J43,K76	1.3 (1.3, 1.3)
1017	I63,N40	1.33 (1.33, 1.33)	1064	J32,K25	1.31 (1.3, 1.32)	1111	H81,N39	1.29 (1.29, 1.3)
1018	G44,M51	1.33 (1.33, 1.33)	1065	I67,N28	1.31 (1.31, 1.31)	1112	K31,M48	1.29 (1.29, 1.3)
1019	J18,M06	1.33 (1.33, 1.33)	1066	K21,M51	1.31 (1.31, 1.31)	1113	D50,J43	1.29 (1.29, 1.3)
1020	E14,K76	1.33 (1.33, 1.33)	1067	E11,K80	1.31 (1.31, 1.31)	1114	J42,J45	1.29 (1.29, 1.3)
1021	H25,N18	1.33 (1.33, 1.33)	1068	E03,I67	1.31 (1.31, 1.31)	1115	I31,I70	1.29 (1.29, 1.29)
1022	I70,J43	1.33 (1.33, 1.33)	1069	H25,I38	1.31 (1.3, 1.32)	1116	G45,K27	1.29 (1.29, 1.3)
1023	K59,M06	1.33 (1.32, 1.34)	1070	K21,N28	1.31 (1.31, 1.31)	1117	I42,N28	1.29 (1.29, 1.29)
1024	H26,M06	1.33 (1.31, 1.34)	1071	I49,N28	1.31 (1.31, 1.31)	1118	I38,N39	1.29 (1.29, 1.3)
1025	I49,K21	1.33 (1.33, 1.33)	1072	I63,N39	1.31 (1.31, 1.31)	1119	D69,K25	1.29 (1.28, 1.3)
1026	G47,N40	1.33 (1.33, 1.33)	1073	E11,E79	1.31 (1.31, 1.31)	1120	I63,M17	1.29 (1.29, 1.29)
1027	E11,H25	1.33 (1.33, 1.33)	1074	H81,K29	1.31 (1.31, 1.31)	1121	H26,H81	1.29 (1.28, 1.3)
1028	I38,K21	1.33 (1.32, 1.33)	1075	E77,I45	1.31 (1.3, 1.31)	1122	J44,K27	1.29 (1.29, 1.29)
1029	I11,M48	1.33 (1.33, 1.33)	1076	I38,K80	1.31 (1.31, 1.31)	1123	K27,K80	1.29 (1.29, 1.29)
1030	G45,K59	1.33 (1.32, 1.33)	1077	K27,M81	1.31 (1.3, 1.31)	1124	K59,R93	2.07 (2.06, 2.08)
1031	G47,I38	1.33 (1.32, 1.33)	1078	G44,K59	1.31 (1.3, 1.32)	1125	K72,R93	2.07 (2.06, 2.08)
1032	I67,I69	1.33 (1.33, 1.33)	1079	J45,K72	1.31 (1.3, 1.31)	1126	J42,K29	1.29 (1.29, 1.29)
1033	I10,I63	1.33 (1.33, 1.33)	1080	J84,M10	1.31 (1.29, 1.32)	1127	K29,M50	1.29 (1.29, 1.29)
1034	K76,N39	1.32 (1.32, 1.33)	1081	J42,M81	1.31 (1.3, 1.31)	1128	J43,K82	1.29 (1.28, 1.29)

TABLE S2. Data in the form of comorbidity network in IHD patients (continued)

ID	Disease pair	OER (99% CI)	ID	Disease pair	OER (99% CI)	ID	Disease pair	OER (99% CI)
1129	J18,N39	1.29 (1.29, 1.29)	1176	E79,I63	1.27 (1.27, 1.27)	1223	I45,I67	1.25 (1.25, 1.25)
1130	H81,M17	1.29 (1.28, 1.29)	1177	J44,K72	1.27 (1.27, 1.27)	1224	D64,K82	1.25 (1.25, 1.25)
1131	I63,N28	1.29 (1.29, 1.29)	1178	J42,M06	1.27 (1.26, 1.27)	1225	I47,I65	1.25 (1.24, 1.25)
1132	I45,N19	1.28 (1.27, 1.3)	1179	K29,M48	1.27 (1.27, 1.27)	1226	I38,J42	1.25 (1.25, 1.25)
1133	I67,M81	1.28 (1.28, 1.28)	1180	E79,I67	1.27 (1.27, 1.27)	1227	I49,N40	1.25 (1.25, 1.25)
1134	H81,M51	1.28 (1.28, 1.29)	1181	G44,K27	1.27 (1.25, 1.28)	1228	J18,N40	1.25 (1.25, 1.25)
1135	I51,K27	1.28 (1.28, 1.29)	1182	E79,M06	1.27 (1.26, 1.27)	1229	D64,M89	1.25 (1.25, 1.25)
1136	K52,K80	1.28 (1.28, 1.28)	1183	G45,N28	1.27 (1.26, 1.27)	1230	I31,J42	1.25 (1.24, 1.25)
1137	I49,M48	1.28 (1.28, 1.28)	1184	I10,N19	1.26 (1.26, 1.27)	1231	J18,K76	1.25 (1.25, 1.25)
1138	K25,K76	1.28 (1.28, 1.28)	1185	D50,K29	1.26 (1.26, 1.26)	1232	G31,N19	1.25 (1.24, 1.25)
1139	I48,K59	1.28 (1.28, 1.28)	1186	G31,K52	1.26 (1.26, 1.27)	1233	K31,N19	1.25 (1.24, 1.25)
1140	I49,K72	1.28 (1.28, 1.28)	1187	I11,K72	1.26 (1.26, 1.27)	1234	E14,M48	1.25 (1.25, 1.25)
1141	E14,E79	1.28 (1.28, 1.28)	1188	G47,N18	1.26 (1.26, 1.27)	1235	D50,I11	1.25 (1.24, 1.25)
1142	E04,H25	1.28 (1.27, 1.29)	1189	I11,M47	1.26 (1.26, 1.26)	1236	I31,J32	1.25 (1.23, 1.26)
1143	E77,K82	1.28 (1.28, 1.28)	1190	K80,R93	1.8 (1.79, 1.8)	1237	I48,J18	1.25 (1.25, 1.25)
1144	I38,I63	1.28 (1.28, 1.28)	1191	I49,J96	1.26 (1.26, 1.26)	1238	K52,M81	1.24 (1.24, 1.25)
1145	M48,N40	1.28 (1.28, 1.28)	1192	K29,M51	1.26 (1.26, 1.26)	1239	I44,I63	1.24 (1.24, 1.25)
1146	G44,K76	1.28 (1.28, 1.28)	1193	I48,I70	1.26 (1.26, 1.26)	1240	I49,K27	1.24 (1.24, 1.25)
1147	F41,M06	1.28 (1.26, 1.29)	1194	G31,K82	1.26 (1.26, 1.26)	1241	E77,I11	1.24 (1.24, 1.24)
1148	H26,I45	1.28 (1.26, 1.3)	1195	I38,M89	1.26 (1.25, 1.27)	1242	M50,N40	1.24 (1.24, 1.24)
1149	I70,N39	1.28 (1.28, 1.28)	1196	G45,I11	1.26 (1.26, 1.26)	1243	D69,K21	1.24 (1.24, 1.25)
1150	K76,R93	2.17 (2.16, 2.17)	1197	E78,N39	1.26 (1.26, 1.26)	1244	E77,H26	1.24 (1.24, 1.25)
1151	I31,N40	1.27 (1.27, 1.28)	1198	E79,J43	1.26 (1.26, 1.26)	1245	I27,N28	1.24 (1.24, 1.24)
1152	I45,J32	1.27 (1.26, 1.29)	1199	K80,M10	1.26 (1.26, 1.26)	1246	J32,K29	1.24 (1.24, 1.24)
1153	G47,M10	1.27 (1.27, 1.28)	1200	E78,N28	1.26 (1.26, 1.26)	1247	D69,H81	1.24 (1.23, 1.25)
1154	K21,M06	1.27 (1.26, 1.28)	1201	E03,G45	1.26 (1.26, 1.26)	1248	D69,G44	1.24 (1.23, 1.25)
1155	J32,K52	1.27 (1.27, 1.28)	1202	K82,R93	2.57 (2.56, 2.59)	1249	I10,I70	1.24 (1.24, 1.24)
1156	I63,N19	1.27 (1.27, 1.27)	1203	J42,K25	1.26 (1.26, 1.26)	1250	I67,K52	1.24 (1.24, 1.24)
1157	I67,J42	1.27 (1.27, 1.27)	1204	K76,M81	1.26 (1.26, 1.26)	1251	K29,K82	1.24 (1.24, 1.24)
1158	D69,I27	1.27 (1.27, 1.27)	1205	J42,M50	1.26 (1.26, 1.26)	1252	I42,J96	1.24 (1.23, 1.25)
1159	J18,J32	1.27 (1.27, 1.27)	1206	H25,N28	1.26 (1.26, 1.26)	1253	J47,K29	1.24 (1.24, 1.24)
1160	J96,K21	1.27 (1.27, 1.28)	1207	E11,I63	1.26 (1.26, 1.26)	1254	E03,H81	1.24 (1.23, 1.25)
1161	E11,E77	1.27 (1.27, 1.27)	1208	I50,J44	1.26 (1.26, 1.26)	1255	D64,K21	1.24 (1.24, 1.24)
1162	J84,K27	1.27 (1.25, 1.29)	1209	J47,M81	1.25 (1.25, 1.26)	1256	D69,J42	1.24 (1.24, 1.24)
1163	I27,K21	1.27 (1.27, 1.27)	1210	D64,I69	1.25 (1.25, 1.25)	1257	D64,E11	1.24 (1.24, 1.24)
1164	E03,I48	1.27 (1.27, 1.27)	1211	D64,I44	1.25 (1.25, 1.26)	1258	E03,M51	1.24 (1.24, 1.24)
1165	I27,I45	1.27 (1.27, 1.27)	1212	D64,I70	1.25 (1.25, 1.25)	1259	K25,N40	1.24 (1.24, 1.24)
1166	I47,K59	1.27 (1.26, 1.28)	1213	J44,N28	1.25 (1.25, 1.25)	1260	I42,K82	1.24 (1.22, 1.25)
1167	I11,J18	1.27 (1.27, 1.27)	1214	I63,M81	1.25 (1.25, 1.25)	1261	G44,N39	1.24 (1.23, 1.24)
1168	E03,E11	1.27 (1.27, 1.27)	1215	I70,M51	1.25 (1.25, 1.25)	1262	E79,M47	1.24 (1.24, 1.24)
1169	J42,J84	1.27 (1.27, 1.27)	1216	I44,K76	1.25 (1.25, 1.25)	1263	D69,I65	1.24 (1.23, 1.24)
1170	F41,I11	1.27 (1.27, 1.27)	1217	E79,I10	1.25 (1.25, 1.25)	1264	E77,I63	1.24 (1.24, 1.24)
1171	D64,J43	1.27 (1.27, 1.27)	1218	I69,K80	1.25 (1.25, 1.25)	1265	H26,I67	1.24 (1.23, 1.24)
1172	E77,I49	1.27 (1.27, 1.27)	1219	E79,H25	1.25 (1.25, 1.25)	1266	E03,K80	1.24 (1.23, 1.24)
1173	G45,K29	1.27 (1.27, 1.27)	1220	D50,E14	1.25 (1.24, 1.26)	1267	E14,J18	1.24 (1.23, 1.24)
1174	H25,J42	1.27 (1.27, 1.27)	1221	I27,J42	1.25 (1.25, 1.25)	1268	K82,N18	1.24 (1.23, 1.24)
1175	E77,J45	1.27 (1.26, 1.27)	1222	K82,M51	1.25 (1.25, 1.25)	1269	I27,K29	1.23 (1.23, 1.23)

TABLE S2. Data in the form of comorbidity network in IHD patients (continued)

ID	Disease pair	OER (99% CI)	ID	Disease pair	OER (99% CI)	ID	Disease pair	OER (99% CI)
1270	E14,I63	1.23 (1.23, 1.23)	1317	G45,K52	1.22 (1.22, 1.22)	1364	I44,K21	1.2 (1.19, 1.21)
1271	J43,N19	1.23 (1.23, 1.24)	1318	I65,M51	1.22 (1.22, 1.22)	1365	I44,N39	1.2 (1.2, 1.21)
1272	I47,J84	1.23 (1.22, 1.25)	1319	H25,I45	1.22 (1.21, 1.23)	1366	E11,I44	1.2 (1.2, 1.2)
1273	G44,H25	1.23 (1.23, 1.24)	1320	E79,K21	1.22 (1.22, 1.22)	1367	E04,I10	1.2 (1.2, 1.2)
1274	I50,J18	1.23 (1.23, 1.23)	1321	I49,J43	1.22 (1.22, 1.22)	1368	I45,I63	1.2 (1.2, 1.2)
1275	H26,J84	1.23 (1.21, 1.25)	1322	I45,J96	1.22 (1.21, 1.22)	1369	J84,M89	1.2 (1.19, 1.22)
1276	J45,K31	1.23 (1.23, 1.24)	1323	I49,J18	1.22 (1.22, 1.22)	1370	I65,K21	1.2 (1.2, 1.2)
1277	E03,J45	1.23 (1.22, 1.25)	1324	E78,K31	1.22 (1.22, 1.22)	1371	I44,J43	1.2 (1.2, 1.2)
1278	H81,K59	1.23 (1.23, 1.24)	1325	H26,I38	1.22 (1.2, 1.23)	1372	J45,N28	1.2 (1.2, 1.2)
1279	K82,N39	1.23 (1.23, 1.24)	1326	I65,M81	1.22 (1.21, 1.22)	1373	K29,M81	1.2 (1.2, 1.2)
1280	G45,N39	1.23 (1.23, 1.23)	1327	E14,M17	1.22 (1.21, 1.22)	1374	E78,I69	1.2 (1.2, 1.2)
1281	M47,N40	1.23 (1.23, 1.23)	1328	D50,K80	1.22 (1.21, 1.22)	1375	G45,N40	1.2 (1.2, 1.2)
1282	I49,K82	1.23 (1.23, 1.23)	1329	I63,M89	1.22 (1.21, 1.22)	1376	K31,M81	1.2 (1.19, 1.2)
1283	D50,I48	1.23 (1.23, 1.23)	1330	I50,M10	1.22 (1.22, 1.22)	1377	J42,J47	1.2 (1.19, 1.2)
1284	E14,M51	1.23 (1.23, 1.23)	1331	G45,K25	1.22 (1.21, 1.22)	1378	H25,I44	1.2 (1.19, 1.2)
1285	E14,I70	1.23 (1.23, 1.23)	1332	K29,K52	1.22 (1.21, 1.22)	1379	E03,K82	1.2 (1.18, 1.21)
1286	I47,N28	1.23 (1.23, 1.23)	1333	E04,N19	1.21 (1.2, 1.22)	1380	G31,J44	1.2 (1.2, 1.2)
1287	M51,N28	1.23 (1.23, 1.23)	1334	G47,J84	1.21 (1.2, 1.23)	1381	I49,I63	1.2 (1.2, 1.2)
1288	I38,M06	1.23 (1.22, 1.24)	1335	E04,I31	1.21 (1.2, 1.23)	1382	H25,M10	1.19 (1.19, 1.2)
1289	G45,M17	1.23 (1.23, 1.23)	1336	K31,M10	1.21 (1.21, 1.22)	1383	K82,N19	1.19 (1.18, 1.2)
1290	I69,N28	1.23 (1.23, 1.23)	1337	H26,K21	1.21 (1.2, 1.22)	1384	G45,K31	1.19 (1.19, 1.2)
1291	E14,K59	1.23 (1.22, 1.23)	1338	J47,M89	1.21 (1.21, 1.22)	1385	I11,M81	1.19 (1.19, 1.19)
1292	K31,M50	1.23 (1.22, 1.23)	1339	D50,J84	1.21 (1.19, 1.23)	1386	H25,M48	1.19 (1.19, 1.2)
1293	K80,N40	1.23 (1.23, 1.23)	1340	E14,J32	1.21 (1.21, 1.22)	1387	E03,K52	1.19 (1.19, 1.2)
1294	J84,K31	1.23 (1.22, 1.24)	1341	D69,I50	1.21 (1.21, 1.21)	1388	H81,K76	1.19 (1.19, 1.19)
1295	J32,M81	1.23 (1.22, 1.23)	1342	K21,N40	1.21 (1.21, 1.21)	1389	I49,K76	1.19 (1.19, 1.19)
1296	E03,N28	1.23 (1.22, 1.23)	1343	J18,K52	1.21 (1.21, 1.21)	1390	J43,M81	1.19 (1.19, 1.19)
1297	D69,G45	1.23 (1.22, 1.23)	1344	D64,J47	1.21 (1.21, 1.21)	1391	E78,I10	1.19 (1.19, 1.19)
1298	H26,I10	1.23 (1.23, 1.23)	1345	E14,K72	1.21 (1.2, 1.21)	1392	H25,I67	1.19 (1.19, 1.19)
1299	I27,I49	1.22 (1.22, 1.22)	1346	K31,M47	1.21 (1.21, 1.21)	1393	H81,I69	1.19 (1.19, 1.2)
1300	G47,I42	1.22 (1.21, 1.23)	1347	J84,N39	1.21 (1.2, 1.22)	1394	E78,G31	1.19 (1.19, 1.19)
1301	J47,K76	1.22 (1.22, 1.22)	1348	G31,K80	1.21 (1.21, 1.21)	1395	I49,N18	1.19 (1.19, 1.19)
1302	E14,J96	1.22 (1.22, 1.22)	1349	I49,I67	1.21 (1.21, 1.21)	1396	I63,K76	1.19 (1.19, 1.19)
1303	I11,N39	1.22 (1.22, 1.22)	1350	I11,M50	1.21 (1.21, 1.21)	1397	I63,M51	1.19 (1.19, 1.19)
1304	J18,K80	1.22 (1.22, 1.22)	1351	I63,N18	1.21 (1.21, 1.21)	1398	J84,K25	1.19 (1.17, 1.21)
1305	E77,K21	1.22 (1.22, 1.22)	1352	E78,N18	1.21 (1.21, 1.21)	1399	K31,K80	1.19 (1.19, 1.19)
1306	H81,K27	1.22 (1.21, 1.23)	1353	M10,R93	1.54 (1.53, 1.55)	1400	E79,M50	1.19 (1.19, 1.19)
1307	E77,I47	1.22 (1.22, 1.22)	1354	I48,N18	1.21 (1.21, 1.21)	1401	I69,M17	1.19 (1.19, 1.19)
1308	I50,K27	1.22 (1.22, 1.22)	1355	G47,K27	1.21 (1.19, 1.22)	1402	E78,K72	1.19 (1.19, 1.19)
1309	M06,R93	1.1 (1.09, 1.12)	1356	F41,N28	1.21 (1.2, 1.21)	1403	I27,I47	1.19 (1.19, 1.19)
1310	M10,M81	1.22 (1.22, 1.22)	1357	J32,N39	1.21 (1.2, 1.21)	1404	E78,I49	1.19 (1.19, 1.19)
1311	G31,I49	1.22 (1.22, 1.22)	1358	H25,N19	1.2 (1.2, 1.21)	1405	E79,K25	1.19 (1.19, 1.19)
1312	H25,J84	1.22 (1.21, 1.23)	1359	F41,J84	1.2 (1.19, 1.22)	1406	I11,J84	1.19 (1.19, 1.19)
1313	I70,J18	1.22 (1.22, 1.22)	1360	J45,M81	1.2 (1.2, 1.21)	1407	I63,J18	1.19 (1.19, 1.19)
1314	M06,N18	1.22 (1.21, 1.23)	1361	K72,N40	1.2 (1.2, 1.2)	1408	D64,I45	1.19 (1.18, 1.19)
1315	I38,K76	1.22 (1.22, 1.22)	1362	E03,G44	1.2 (1.19, 1.22)	1409	K52,M17	1.19 (1.18, 1.19)
1316	I48,I63	1.22 (1.22, 1.22)	1363	E03,I50	1.2 (1.2, 1.2)	1410	I69,J42	1.19 (1.19, 1.19)

TABLE S2. Data in the form of comorbidity network in IHD patients (continued)

ID	Disease pair	OER (99% CI)	ID	Disease pair	OER (99% CI)	ID	Disease pair	OER (99% CI)
1411	I47,K76	1.19 (1.19, 1.19)	1458	H25,N39	1.17 (1.17, 1.17)	1505	H81,I11	1.16 (1.16, 1.16)
1412	I69,K27	1.19 (1.18, 1.19)	1459	I42,J42	1.17 (1.17, 1.18)	1506	H25,J45	1.16 (1.15, 1.17)
1413	I50,J84	1.19 (1.18, 1.19)	1460	I48,N28	1.17 (1.17, 1.17)	1507	I65,J43	1.16 (1.16, 1.16)
1414	J18,K82	1.18 (1.18, 1.19)	1461	E78,K80	1.17 (1.17, 1.17)	1508	H26,K52	1.16 (1.15, 1.16)
1415	E14,J45	1.18 (1.18, 1.19)	1462	I63,J42	1.17 (1.17, 1.17)	1509	I47,J43	1.16 (1.15, 1.16)
1416	K25,N39	1.18 (1.18, 1.19)	1463	I50,K59	1.17 (1.17, 1.17)	1510	D64,E04	1.16 (1.15, 1.16)
1417	I47,K21	1.18 (1.18, 1.19)	1464	K52,N40	1.17 (1.17, 1.17)	1511	D69,I67	1.16 (1.16, 1.16)
1418	E79,G45	1.18 (1.18, 1.18)	1465	I44,J18	1.17 (1.17, 1.17)	1512	M51,R93	1.22 (1.22, 1.22)
1419	K82,M10	1.18 (1.18, 1.19)	1466	H25,I10	1.17 (1.17, 1.17)	1513	I45,J18	1.16 (1.15, 1.16)
1420	I48,K27	1.18 (1.18, 1.19)	1467	J18,M10	1.17 (1.17, 1.17)	1514	M47,N39	1.15 (1.15, 1.16)
1421	I65,K80	1.18 (1.18, 1.18)	1468	E03,I63	1.17 (1.17, 1.17)	1515	M81,R93	1.35 (1.34, 1.35)
1422	M50,N39	1.18 (1.18, 1.19)	1469	I50,I70	1.17 (1.17, 1.17)	1516	H81,J42	1.15 (1.15, 1.16)
1423	E11,M81	1.18 (1.18, 1.18)	1470	J32,M06	1.17 (1.15, 1.18)	1517	I51,K76	1.15 (1.15, 1.15)
1424	I67,N39	1.18 (1.18, 1.18)	1471	H25,I11	1.17 (1.17, 1.17)	1518	J18,M81	1.15 (1.15, 1.16)
1425	H26,K80	1.18 (1.18, 1.19)	1472	G45,I49	1.17 (1.17, 1.17)	1519	E78,I47	1.15 (1.15, 1.15)
1426	I44,M50	1.18 (1.17, 1.19)	1473	M48,R93	1.67 (1.67, 1.68)	1520	H81,M81	1.15 (1.15, 1.16)
1427	D69,E14	1.18 (1.18, 1.19)	1474	I38,I69	1.17 (1.16, 1.17)	1521	I48,J44	1.15 (1.15, 1.15)
1428	I31,I47	1.18 (1.17, 1.19)	1475	G45,H25	1.17 (1.17, 1.17)	1522	J45,K29	1.15 (1.15, 1.15)
1429	I42,K76	1.18 (1.18, 1.18)	1476	J42,N39	1.17 (1.17, 1.17)	1523	I42,K31	1.15 (1.14, 1.16)
1430	I11,I42	1.18 (1.18, 1.18)	1477	K82,M06	1.17 (1.15, 1.18)	1524	I69,K72	1.15 (1.15, 1.16)
1431	H26,J18	1.18 (1.18, 1.18)	1478	I31,K76	1.17 (1.16, 1.17)	1525	E78,K52	1.15 (1.15, 1.15)
1432	M17,R93	1.66 (1.65, 1.67)	1479	E14,K82	1.17 (1.16, 1.17)	1526	G45,I45	1.15 (1.15, 1.16)
1433	D69,F41	1.18 (1.17, 1.19)	1480	E79,M81	1.17 (1.16, 1.17)	1527	J96,K80	1.15 (1.15, 1.15)
1434	H26,I44	1.18 (1.17, 1.19)	1481	J43,K21	1.16 (1.16, 1.17)	1528	J44,N19	1.15 (1.15, 1.15)
1435	M47,N28	1.18 (1.18, 1.18)	1482	I49,M47	1.16 (1.16, 1.17)	1529	D69,M06	1.15 (1.14, 1.16)
1436	M81,N18	1.18 (1.18, 1.18)	1483	I70,K72	1.16 (1.16, 1.17)	1530	I49,K80	1.15 (1.15, 1.15)
1437	I65,J45	1.18 (1.17, 1.18)	1484	J84,K80	1.16 (1.16, 1.17)	1531	D64,I27	1.15 (1.15, 1.15)
1438	I49,M10	1.18 (1.18, 1.18)	1485	I51,J43	1.16 (1.16, 1.16)	1532	I47,K82	1.15 (1.14, 1.16)
1439	E03,M89	1.18 (1.17, 1.19)	1486	I31,M10	1.16 (1.15, 1.17)	1533	J84,K52	1.15 (1.14, 1.16)
1440	I42,I70	1.18 (1.18, 1.18)	1487	I49,J47	1.16 (1.16, 1.16)	1534	J45,N40	1.15 (1.15, 1.15)
1441	H26,J42	1.18 (1.17, 1.18)	1488	F41,H25	1.16 (1.16, 1.17)	1535	H26,J45	1.15 (1.13, 1.17)
1442	J18,K21	1.18 (1.17, 1.18)	1489	E79,M51	1.16 (1.16, 1.16)	1536	I45,M17	1.15 (1.14, 1.16)
1443	G44,N28	1.18 (1.17, 1.18)	1490	I38,K29	1.16 (1.16, 1.16)	1537	I51,K80	1.15 (1.15, 1.15)
1444	G47,J42	1.18 (1.17, 1.18)	1491	J42,K27	1.16 (1.16, 1.17)	1538	I51,K82	1.15 (1.15, 1.15)
1445	H26,I69	1.18 (1.17, 1.18)	1492	I70,J47	1.16 (1.16, 1.16)	1539	I49,K52	1.15 (1.15, 1.15)
1446	I31,K52	1.17 (1.17, 1.18)	1493	J44,K29	1.16 (1.16, 1.16)	1540	I10,I44	1.15 (1.15, 1.15)
1447	I45,I69	1.17 (1.17, 1.18)	1494	I47,J47	1.16 (1.15, 1.17)	1541	I50,J47	1.15 (1.15, 1.15)
1448	H25,I63	1.17 (1.17, 1.17)	1495	J42,N28	1.16 (1.16, 1.16)	1542	E78,N19	1.15 (1.15, 1.15)
1449	E03,E14	1.17 (1.17, 1.18)	1496	D64,I48	1.16 (1.16, 1.16)	1543	I45,J47	1.15 (1.14, 1.16)
1450	E03,K31	1.17 (1.17, 1.18)	1497	I44,I67	1.16 (1.16, 1.16)	1544	G31,I10	1.15 (1.15, 1.15)
1451	E11,G47	1.17 (1.17, 1.17)	1498	E14,G31	1.16 (1.16, 1.16)	1545	G31,K29	1.15 (1.15, 1.15)
1452	H81,K21	1.17 (1.17, 1.18)	1499	H25,N40	1.16 (1.16, 1.16)	1546	I31,M06	1.15 (1.13, 1.16)
1453	E78,H25	1.17 (1.17, 1.17)	1500	G44,M81	1.16 (1.16, 1.16)	1547	I69,K52	1.15 (1.14, 1.15)
1454	M47,R93	1.35 (1.35, 1.35)	1501	M50,R93	1.48 (1.47, 1.49)	1548	I44,K27	1.15 (1.13, 1.16)
1455	I44,M48	1.17 (1.17, 1.18)	1502	G47,I10	1.16 (1.16, 1.16)	1549	I42,J45	1.15 (1.13, 1.16)
1456	E04,J43	1.17 (1.17, 1.18)	1503	I10,M17	1.16 (1.16, 1.16)	1550	I10,K76	1.15 (1.15, 1.15)
1457	I42,J84	1.17 (1.15, 1.19)	1504	D69,I11	1.16 (1.16, 1.16)	1551	I42,J43	1.15 (1.14, 1.15)

TABLE S2. Data in the form of comorbidity network in IHD patients (continued)

ID	Disease pair	OER (99% CI)	ID	Disease pair	OER (99% CI)	ID	Disease pair	OER (99% CI)
1552	D50,J47	1.15 (1.14, 1.16)	1599	H25,M51	1.13 (1.13, 1.13)	1646	I69,M47	1.11 (1.11, 1.12)
1553	I31,N39	1.15 (1.14, 1.15)	1600	J42,K82	1.13 (1.13, 1.13)	1647	E78,K29	1.11 (1.11, 1.11)
1554	I49,M50	1.15 (1.14, 1.15)	1601	I11,K52	1.13 (1.13, 1.13)	1648	J43,K52	1.11 (1.11, 1.12)
1555	K31,M51	1.15 (1.14, 1.15)	1602	H25,J43	1.13 (1.13, 1.13)	1649	I42,J44	1.11 (1.11, 1.11)
1556	E04,N40	1.15 (1.14, 1.15)	1603	F41,I45	1.13 (1.11, 1.14)	1650	I10,K80	1.11 (1.11, 1.11)
1557	I50,J43	1.15 (1.14, 1.15)	1604	E04,K72	1.13 (1.12, 1.14)	1651	I10,N28	1.11 (1.11, 1.11)
1558	I51,K59	1.14 (1.14, 1.15)	1605	I67,K25	1.13 (1.13, 1.13)	1652	I63,K80	1.11 (1.11, 1.11)
1559	I51,N40	1.14 (1.14, 1.14)	1606	H25,M50	1.13 (1.12, 1.13)	1653	H25,K82	1.11 (1.11, 1.12)
1560	J43,K29	1.14 (1.14, 1.14)	1607	H25,I49	1.13 (1.12, 1.13)	1654	I49,K31	1.11 (1.11, 1.11)
1561	I45,K21	1.14 (1.13, 1.15)	1608	G47,M06	1.13 (1.12, 1.14)	1655	H81,K82	1.11 (1.1, 1.12)
1562	K27,N40	1.14 (1.14, 1.15)	1609	D69,I63	1.13 (1.13, 1.13)	1656	I70,K31	1.11 (1.11, 1.11)
1563	D50,I49	1.14 (1.14, 1.14)	1610	I11,J96	1.13 (1.12, 1.13)	1657	G47,K72	1.11 (1.11, 1.12)
1564	M51,N40	1.14 (1.14, 1.14)	1611	D64,K29	1.12 (1.12, 1.12)	1658	I44,J42	1.11 (1.11, 1.11)
1565	E11,M17	1.14 (1.14, 1.14)	1612	E79,J47	1.12 (1.12, 1.13)	1659	I11,J42	1.11 (1.11, 1.11)
1566	E11,K59	1.14 (1.14, 1.14)	1613	D50,M89	1.12 (1.11, 1.14)	1660	H25,K76	1.11 (1.11, 1.11)
1567	D64,H25	1.14 (1.14, 1.14)	1614	M10,N39	1.12 (1.12, 1.13)	1661	I69,M89	1.11 (1.1, 1.11)
1568	I38,I67	1.14 (1.14, 1.14)	1615	J43,K80	1.12 (1.12, 1.12)	1662	I70,J45	1.11 (1.11, 1.11)
1569	I69,M48	1.14 (1.14, 1.14)	1616	E04,K25	1.12 (1.11, 1.14)	1663	J47,N19	1.11 (1.1, 1.12)
1570	G31,K72	1.14 (1.14, 1.14)	1617	K82,M81	1.12 (1.12, 1.13)	1664	K29,N39	1.11 (1.11, 1.11)
1571	E77,K76	1.14 (1.14, 1.14)	1618	I69,J96	1.12 (1.12, 1.12)	1665	K29,K76	1.11 (1.11, 1.11)
1572	I45,N39	1.14 (1.13, 1.15)	1619	E14,G47	1.12 (1.12, 1.13)	1666	I42,K80	1.11 (1.1, 1.11)
1573	E77,M89	1.14 (1.14, 1.14)	1620	M10,M48	1.12 (1.12, 1.12)	1667	I45,M47	1.11 (1.1, 1.11)
1574	I45,J42	1.14 (1.14, 1.14)	1621	K76,N19	1.12 (1.12, 1.12)	1668	I51,I65	1.11 (1.11, 1.11)
1575	M89,R93	1.52 (1.51, 1.54)	1622	E78,I45	1.12 (1.12, 1.12)	1669	H25,K21	1.11 (1.1, 1.11)
1576	D50,I27	1.14 (1.14, 1.14)	1623	I48,K80	1.12 (1.12, 1.12)	1670	G31,M06	1.11 (1.1, 1.11)
1577	G45,I38	1.14 (1.14, 1.14)	1624	I48,I65	1.12 (1.12, 1.12)	1671	E11,K82	1.11 (1.11, 1.11)
1578	M10,M89	1.14 (1.13, 1.15)	1625	I49,N39	1.12 (1.12, 1.12)	1672	I31,K31	1.11 (1.1, 1.11)
1579	I45,M81	1.14 (1.13, 1.14)	1626	I45,M51	1.12 (1.12, 1.12)	1673	N19,R93	1.92 (1.9, 1.93)
1580	J44,K21	1.14 (1.14, 1.14)	1627	I69,M51	1.12 (1.12, 1.12)	1674	D64,M48	1.1 (1.1, 1.11)
1581	J45,M48	1.14 (1.13, 1.14)	1628	H81,K25	1.12 (1.11, 1.13)	1675	E79,K31	1.1 (1.1, 1.11)
1582	H26,I49	1.14 (1.13, 1.14)	1629	E14,M06	1.12 (1.11, 1.13)	1676	I50,J45	1.1 (1.1, 1.1)
1583	I49,J45	1.14 (1.13, 1.14)	1630	I42,K52	1.12 (1.11, 1.13)	1677	I47,M10	1.1 (1.1, 1.11)
1584	I31,K25	1.14 (1.12, 1.15)	1631	I11,M89	1.12 (1.12, 1.12)	1678	I11,M51	1.1 (1.1, 1.1)
1585	K80,M81	1.14 (1.13, 1.14)	1632	J96,K31	1.12 (1.11, 1.12)	1679	K21,N19	1.1 (1.1, 1.11)
1586	K29,K72	1.14 (1.13, 1.14)	1633	D69,J47	1.12 (1.11, 1.12)	1680	J45,M06	1.1 (1.09, 1.12)
1587	K80,M89	1.13 (1.13, 1.14)	1634	H81,I49	1.12 (1.12, 1.12)	1681	E77,H25	1.1 (1.1, 1.11)
1588	I44,K82	1.13 (1.12, 1.14)	1635	J45,M47	1.12 (1.11, 1.12)	1682	G31,N18	1.1 (1.1, 1.1)
1589	E78,I44	1.13 (1.13, 1.13)	1636	I42,N40	1.12 (1.11, 1.12)	1683	I49,M81	1.1 (1.1, 1.1)
1590	J96,K82	1.13 (1.13, 1.14)	1637	G45,I10	1.12 (1.12, 1.12)	1684	I11,K27	1.1 (1.1, 1.1)
1591	I47,I67	1.13 (1.13, 1.13)	1638	I47,K31	1.12 (1.11, 1.12)	1685	I47,J45	1.1 (1.09, 1.11)
1592	I51,J44	1.13 (1.13, 1.13)	1639	K29,M17	1.12 (1.11, 1.12)	1686	M10,M51	1.1 (1.1, 1.1)
1593	D64,I49	1.13 (1.13, 1.13)	1640	I49,J32	1.12 (1.11, 1.12)	1687	G45,J42	1.1 (1.1, 1.1)
1594	E04,E77	1.13 (1.13, 1.13)	1641	D69,M47	1.12 (1.11, 1.12)	1688	H25,M47	1.1 (1.1, 1.1)
1595	D64,G47	1.13 (1.13, 1.13)	1642	K21,K80	1.12 (1.11, 1.12)	1689	N28,R93	2.94 (2.94, 2.95)
1596	K72,M06	1.13 (1.12, 1.14)	1643	H26,J47	1.12 (1.11, 1.12)	1690	H25,H81	1.1 (1.09, 1.1)
1597	N18,R93	1.63 (1.62, 1.63)	1644	I50,N28	1.11 (1.11, 1.12)	1691	I47,J32	1.1 (1.09, 1.11)
1598	I69,J84	1.13 (1.12, 1.14)	1645	E11,K72	1.11 (1.11, 1.12)	1692	G47,K80	1.1 (1.1, 1.1)

TABLE S2. Data in the form of comorbidity network in IHD patients (continued)

ID	Disease pair	OER (99% CI)	ID	Disease pair	OER (99% CI)	ID	Disease pair	OER (99% CI)
1693	I63,K82	1.1 (1.1, 1.1)	1740	I67,K80	1.08 (1.08, 1.08)	1787	I50,I69	1.06 (1.06, 1.07)
1694	D64,I63	1.1 (1.1, 1.1)	1741	I63,M10	1.08 (1.08, 1.08)	1788	E79,J42	1.06 (1.06, 1.07)
1695	J47,K80	1.1 (1.09, 1.1)	1742	D69,K29	1.08 (1.08, 1.08)	1789	J84,M17	1.06 (1.05, 1.08)
1696	K29,N28	1.1 (1.1, 1.1)	1743	I65,M89	1.08 (1.08, 1.09)	1790	I10,I51	1.06 (1.06, 1.06)
1697	G31,K25	1.1 (1.09, 1.1)	1744	I11,K25	1.08 (1.08, 1.08)	1791	K25,K80	1.06 (1.06, 1.06)
1698	J45,K27	1.1 (1.08, 1.11)	1745	H25,K27	1.08 (1.07, 1.09)	1792	E03,I10	1.06 (1.06, 1.06)
1699	D50,N40	1.1 (1.09, 1.1)	1746	F41,K27	1.08 (1.07, 1.1)	1793	H25,I48	1.06 (1.06, 1.06)
1700	J45,K80	1.1 (1.09, 1.1)	1747	E14,I67	1.08 (1.08, 1.08)	1794	D50,G44	1.06 (1.04, 1.08)
1701	G45,I44	1.09 (1.09, 1.1)	1748	I49,J44	1.08 (1.08, 1.08)	1795	I42,K29	1.06 (1.06, 1.06)
1702	K80,M48	1.09 (1.09, 1.09)	1749	D69,I69	1.08 (1.08, 1.08)	1796	G47,J18	1.06 (1.06, 1.06)
1703	G44,J42	1.09 (1.09, 1.1)	1750	E11,I67	1.08 (1.08, 1.08)	1797	I10,M47	1.06 (1.06, 1.06)
1704	K80,M06	1.09 (1.09, 1.1)	1751	E79,F41	1.08 (1.08, 1.08)	1798	D64,M17	1.06 (1.06, 1.06)
1705	J43,K31	1.09 (1.09, 1.1)	1752	G47,J47	1.08 (1.07, 1.08)	1799	J32,K80	1.06 (1.06, 1.06)
1706	J47,K25	1.09 (1.09, 1.1)	1753	E11,K52	1.08 (1.08, 1.08)	1800	M81,N19	1.06 (1.05, 1.06)
1707	D64,J44	1.09 (1.09, 1.09)	1754	E77,M48	1.08 (1.08, 1.08)	1801	I27,K31	1.06 (1.06, 1.06)
1708	I49,I69	1.09 (1.09, 1.09)	1755	J18,M89	1.08 (1.07, 1.08)	1802	J42,K31	1.05 (1.05, 1.06)
1709	E77,J42	1.09 (1.09, 1.09)	1756	J45,M17	1.08 (1.07, 1.09)	1803	J44,M81	1.05 (1.05, 1.05)
1710	G45,I47	1.09 (1.09, 1.09)	1757	D50,I44	1.08 (1.06, 1.09)	1804	G31,I51	1.05 (1.05, 1.06)
1711	I31,J45	1.09 (1.08, 1.11)	1758	E03,G31	1.08 (1.07, 1.08)	1805	J18,K29	1.05 (1.05, 1.05)
1712	I11,J45	1.09 (1.09, 1.09)	1759	E79,J44	1.08 (1.08, 1.08)	1806	I10,I49	1.05 (1.05, 1.05)
1713	D64,N40	1.09 (1.09, 1.09)	1760	E04,K29	1.08 (1.08, 1.08)	1807	I65,M17	1.05 (1.05, 1.06)
1714	I49,J42	1.09 (1.09, 1.09)	1761	I48,N40	1.08 (1.08, 1.08)	1808	J18,K25	1.05 (1.05, 1.06)
1715	H25,J47	1.09 (1.08, 1.09)	1762	D69,J44	1.07 (1.07, 1.08)	1809	E11,J18	1.05 (1.05, 1.05)
1716	K21,M10	1.09 (1.08, 1.09)	1763	E11,N28	1.07 (1.07, 1.07)	1810	I47,N40	1.05 (1.05, 1.05)
1717	I45,J44	1.09 (1.09, 1.09)	1764	I10,K59	1.07 (1.07, 1.07)	1811	E77,I65	1.05 (1.05, 1.05)
1718	E77,J32	1.09 (1.08, 1.09)	1765	E14,J84	1.07 (1.06, 1.08)	1812	E11,G45	1.05 (1.05, 1.05)
1719	I10,N39	1.09 (1.09, 1.09)	1766	I49,K25	1.07 (1.07, 1.07)	1813	G44,J47	1.05 (1.04, 1.06)
1720	H26,J43	1.09 (1.08, 1.09)	1767	I47,J18	1.07 (1.07, 1.08)	1814	I44,K31	1.05 (1.04, 1.06)
1721	I45,K27	1.09 (1.07, 1.11)	1768	E78,K25	1.07 (1.07, 1.07)	1815	I63,K52	1.05 (1.05, 1.05)
1722	K27,K76	1.09 (1.09, 1.09)	1769	I10,M81	1.07 (1.07, 1.07)	1816	I69,K76	1.05 (1.05, 1.05)
1723	J45,M89	1.09 (1.08, 1.1)	1770	I10,N40	1.07 (1.07, 1.07)	1817	I31,I69	1.05 (1.04, 1.05)
1724	E03,J18	1.09 (1.08, 1.09)	1771	E14,N28	1.07 (1.07, 1.07)	1818	J18,K31	1.05 (1.05, 1.05)
1725	I49,M17	1.09 (1.09, 1.09)	1772	I51,J47	1.07 (1.07, 1.07)	1819	G47,I51	1.05 (1.05, 1.05)
1726	F41,I38	1.09 (1.08, 1.1)	1773	J45,K52	1.07 (1.06, 1.08)	1820	I63,K21	1.05 (1.05, 1.05)
1727	I51,I69	1.09 (1.08, 1.09)	1774	I10,J32	1.07 (1.07, 1.07)	1821	I44,J32	1.05 (1.04, 1.06)
1728	K31,N18	1.09 (1.08, 1.09)	1775	I42,K59	1.07 (1.06, 1.08)	1822	I69,K82	1.05 (1.04, 1.05)
1729	J42,K80	1.08 (1.08, 1.09)	1776	I63,J43	1.07 (1.07, 1.07)	1823	E03,K29	1.05 (1.05, 1.05)
1730	D50,J96	1.08 (1.08, 1.09)	1777	K27,K29	1.07 (1.07, 1.07)	1824	N39,R93	1.92 (1.91, 1.93)
1731	I63,K27	1.08 (1.08, 1.09)	1778	I44,M17	1.07 (1.06, 1.08)	1825	I10,M50	1.05 (1.05, 1.05)
1732	I44,J96	1.08 (1.08, 1.09)	1779	I10,M48	1.07 (1.07, 1.07)	1826	M06,N28	1.05 (1.04, 1.05)
1733	I49,M89	1.08 (1.08, 1.09)	1780	H81,M06	1.07 (1.06, 1.08)	1827	G31,K21	1.05 (1.04, 1.05)
1734	J42,K72	1.08 (1.08, 1.09)	1781	I45,K52	1.07 (1.06, 1.07)	1828	I10,K82	1.05 (1.05, 1.05)
1735	I10,I67	1.08 (1.08, 1.08)	1782	I50,N40	1.07 (1.07, 1.07)	1829	D50,E04	1.05 (1.03, 1.06)
1736	I38,M17	1.08 (1.08, 1.09)	1783	E14,N40	1.07 (1.06, 1.07)	1830	I48,J43	1.05 (1.04, 1.05)
1737	I70,K52	1.08 (1.08, 1.08)	1784	D64,J42	1.07 (1.06, 1.07)	1831	K27,M51	1.04 (1.04, 1.05)
1738	D69,M17	1.08 (1.08, 1.09)	1785	J32,K27	1.07 (1.05, 1.08)	1832	I44,M81	1.04 (1.04, 1.05)
1739	J42,K76	1.08 (1.08, 1.08)	1786	J84,K29	1.07 (1.06, 1.07)	1833	I47,K29	1.04 (1.04, 1.05)

TABLE S2. Data in the form of comorbidity network in IHD patients (continued)

ID	Disease pair	OER (99% CI)	ID	Disease pair	OER (99% CI)	ID	Disease pair	OER (99% CI)
1834	G44,J45	1.04 (1.03, 1.06)	1866	I42,K21	1.03 (1.02, 1.04)	1898	E11,I50	1.02 (1.02, 1.02)
1835	H81,I10	1.04 (1.04, 1.04)	1867	I47,K80	1.03 (1.03, 1.03)	1899	I47,M89	1.02 (1.01, 1.03)
1836	I42,I65	1.04 (1.04, 1.05)	1868	E11,J32	1.03 (1.03, 1.03)	1900	J96,M06	1.02 (1.01, 1.02)
1837	E03,I69	1.04 (1.04, 1.05)	1869	I48,N39	1.03 (1.03, 1.03)	1901	E11,J45	1.02 (1.02, 1.02)
1838	H25,M89	1.04 (1.04, 1.05)	1870	J96,K29	1.03 (1.03, 1.03)	1902	J44,K31	1.02 (1.02, 1.02)
1839	I70,K29	1.04 (1.04, 1.04)	1871	I31,M89	1.03 (1.02, 1.04)	1903	I47,K52	1.02 (1.01, 1.02)
1840	J45,M51	1.04 (1.04, 1.04)	1872	I47,J44	1.03 (1.03, 1.03)	1904	E14,I51	1.02 (1.02, 1.02)
1841	I48,J84	1.04 (1.04, 1.05)	1873	I42,K25	1.03 (1.01, 1.04)	1906	G31,I47	1.02 (1.01, 1.02)
1842	F41,I10	1.04 (1.04, 1.04)	1874	G47,N19	1.03 (1.02, 1.03)	1907	J32,K72	1.02 (1.01, 1.02)
1843	I45,K25	1.04 (1.03, 1.06)	1875	I47,N18	1.03 (1.02, 1.03)	1908	E78,M06	1.02 (1.02, 1.02)
1844	J44,K25	1.04 (1.04, 1.04)	1876	I44,M89	1.03 (1.02, 1.04)	1909	I47,M48	1.02 (1.01, 1.02)
1845	G44,I38	1.04 (1.03, 1.05)	1877	I70,J42	1.03 (1.03, 1.03)	1910	J96,K76	1.02 (1.02, 1.02)
1846	D69,E78	1.04 (1.04, 1.04)	1878	I31,I63	1.03 (1.03, 1.03)	1911	I31,K29	1.02 (1.02, 1.02)
1847	I50,K29	1.04 (1.04, 1.04)	1879	E77,G47	1.03 (1.02, 1.03)	1912	E03,K25	1.02 (1.01, 1.03)
1848	H81,I45	1.04 (1.03, 1.05)	1880	I67,M06	1.03 (1.02, 1.03)	1913	E77,K29	1.02 (1.02, 1.02)
1849	I49,K29	1.04 (1.04, 1.04)	1881	I50,I67	1.03 (1.03, 1.03)	1914	N40,R93	1.96 (1.96, 1.96)
1850	I44,K52	1.04 (1.03, 1.04)	1882	J44,M89	1.03 (1.02, 1.03)	1915	K29,N40	1.01 (1.01, 1.01)
1851	E04,I69	1.04 (1.03, 1.04)	1883	G31,J47	1.03 (1.02, 1.03)	1916	E04,I48	1.01 (1.01, 1.02)
1852	E11,N40	1.04 (1.04, 1.04)	1884	I10,I50	1.03 (1.03, 1.03)	1929	D64,J32	1.01 (1, 1.01)
1853	I10,M51	1.04 (1.04, 1.04)	1885	I47,N39	1.02 (1.02, 1.03)	1930	I51,I63	1.01 (1.01, 1.01)
1854	I11,K31	1.04 (1.04, 1.04)	1886	J43,M06	1.02 (1.02, 1.03)	1931	I70,K25	1.01 (1, 1.01)
1855	D50,I70	1.04 (1.04, 1.04)	1887	E11,G31	1.02 (1.02, 1.02)	1932	E14,M50	1.01 (1, 1.01)
1856	E04,I51	1.04 (1.03, 1.04)	1888	K80,M17	1.02 (1.02, 1.03)	1933	I50,J42	1.01 (1, 1.01)
1857	I65,J18	1.04 (1.03, 1.04)	1889	I51,N39	1.02 (1.02, 1.02)	1934	H25,K52	1 (1, 1.01)
1858	G47,I50	1.03 (1.03, 1.03)	1890	I65,K52	1.02 (1.02, 1.03)	1935	D50,J42	1 (1, 1.01)
1859	J45,N19	1.03 (1.02, 1.05)	1891	I27,K25	1.02 (1.02, 1.03)	1936	G44,N40	1 (1, 1.01)
1860	D64,I47	1.03 (1.03, 1.04)	1892	I27,I44	1.02 (1.02, 1.02)	1937	E79,K29	1 (1, 1)
1861	G31,K31	1.03 (1.03, 1.04)	1893	I63,K31	1.02 (1.02, 1.02)	1938	K21,N18	1 (1, 1.01)
1862	I63,K72	1.03 (1.03, 1.03)	1894	D69,E11	1.02 (1.02, 1.02)	1939	J96,N39	1 (1, 1)
1863	E14,I44	1.03 (1.03, 1.04)	1895	I27,K82	1.02 (1.02, 1.02)	1940	G45,K80	1 (1, 1)
1864	I27,K80	1.03 (1.03, 1.03)	1896	I70,J96	1.02 (1.02, 1.02)	1941	E11,I51	1 (1, 1)
1865	I50,K80	1.03 (1.03, 1.03)	1897	J44,K80	1.02 (1.02, 1.02)			

Table S3. Clusters in IHD patients and controls networks

	Clusters	Nodes	Edges	Central disease (ICD-10)	Diseases (ICD-10) within the cluster
IHD patients	A1	34	525	Unspecified kidney failure (N19)	D50, D64, D69, E77, E79, I11, I27, I31, I38, I42, I44, I45, I47, I48, I49, I50, I51, J18, J43, J44, J45, J47, J84, J96, K25, K27, K72, K80, M10, N18, N19, N28, N40, R93
	A2	37	613	Sleep disorders (G47)	E03, E04, E11, E14, E78, F41, G31, G44, G45, G47, H25, H26, H81, I10, I63, I65, I67, I69, I70, J32, J42, K21, K29, K31, K52, K59, K76, K82, M06, M17, M47, M48, M50, M51, M81, M89, N39
Controls	B1	11	48	Respiratory failure, not elsewhere classified (J96)	C34, G93, I27, I61, J18, J42, J43, J44, J47, J96, N40
	B2	9	36	Heart failure (I50)	E79, I11, I38, I48, I49, I50, I51, M10, N18
	B3	24	232	Cervical disc disorders (M50)	E04, E11, E14, E78, G31, G45, G47, H25, H26, H81, I10, I63, I65, I67, I69, I70, J32, M17, M47, M48, M50, M51, M81, M89
	B4	19	162	Hepatic failure, not elsewhere classified (K72)	D50, D64, D69, E77, I84, K21, K25, K29, K31, K52, K59, K72, K74, K76, K80, K82, K83, N28, N39

5 Sex- and Age-specific comorbidity networks

Clusters in sex- and age-specific comorbidity networks in IHD patients are shown in Figure S3, the corresponding central disease in each cluster is shown in Figure S4 and all comorbidities in each cluster are presented in Table S4.

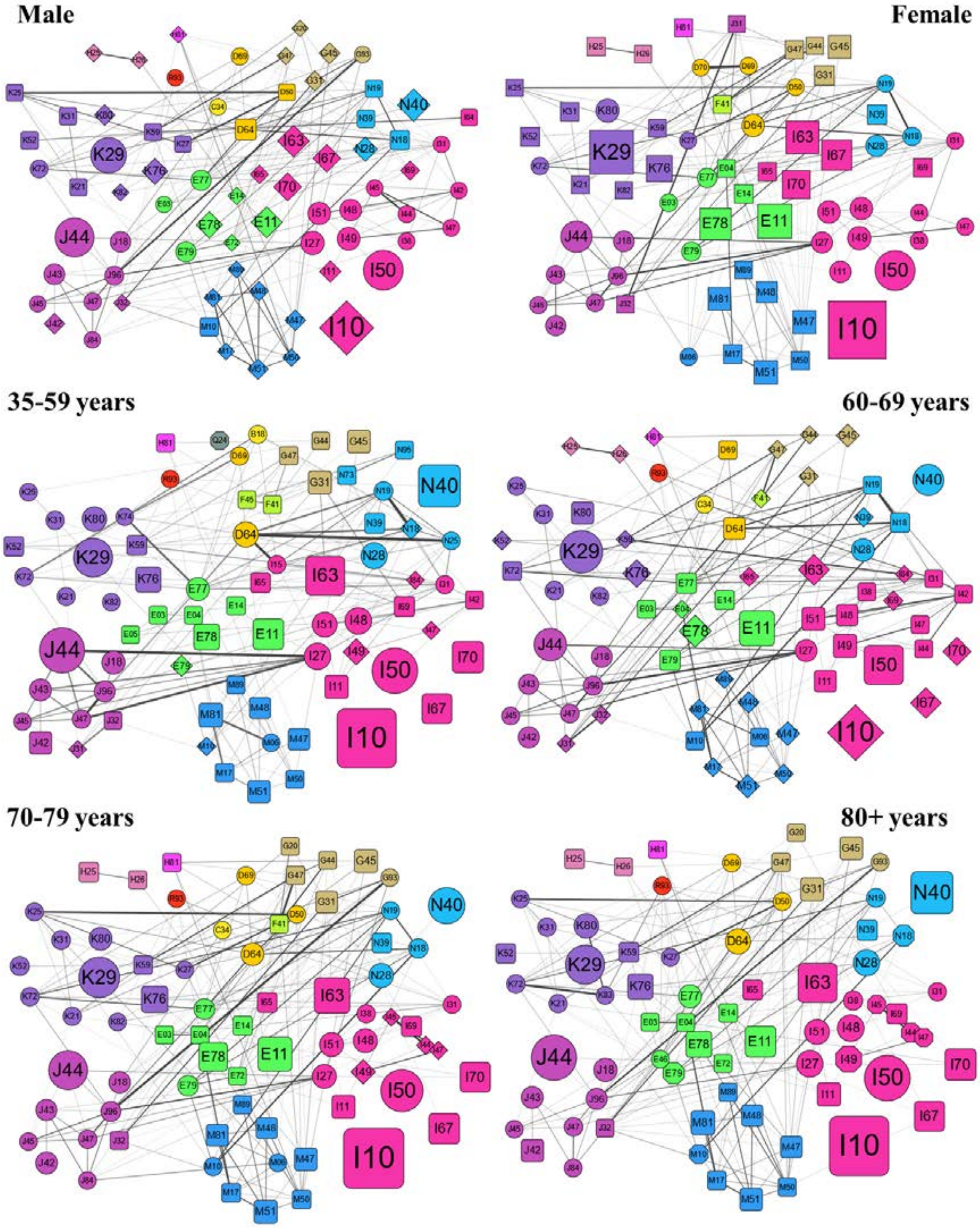

Figure S3. Sex - and age-specific comorbidity networks in IHD patients. Node size represents prevalence, color indicates different disease chapters, and shape denotes different clusters. Edge thickness represents OER value.Here all statistically significant links where OERs>P90 are shown.

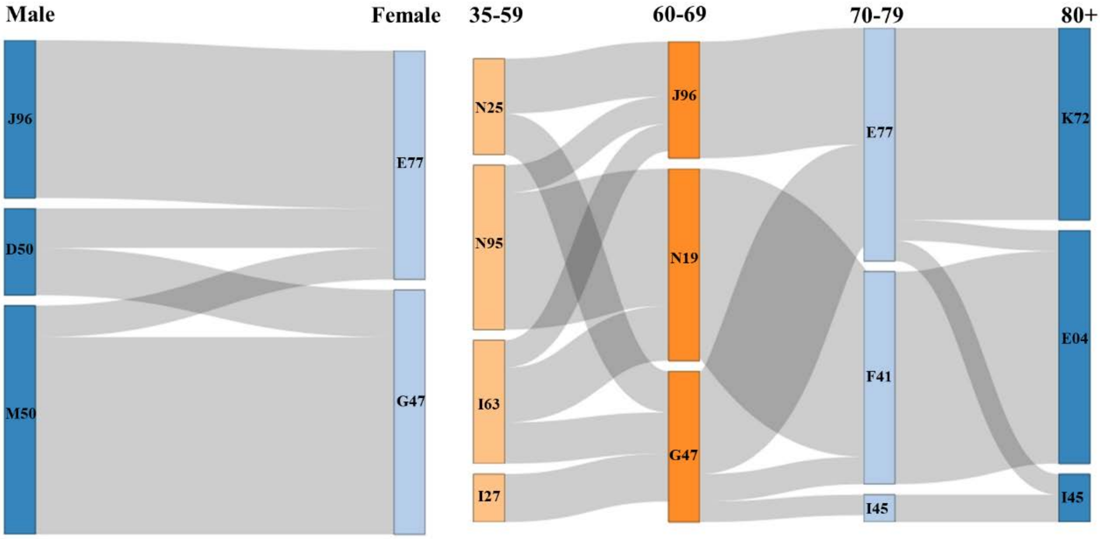

Figure S4. Sex- and age-specific network clustering mulberry graph. Each bar in each column represents a cluster, and the disease in the bar represents the central disease of the cluster.

Table S4. Clusters in sex- and age-specific networks in IHD patients

	Cluster	Nodes	Edges	Central disease (ICD-10)	Diseases (ICD-10) within the cluster
Male	A1	27	316	Respiratory failure, not elsewhere classified (J96)	C34, D69, E03, E77, E79, G93, I27, I31, I38, I42, I44, I45, I47, I48, I49, I50, I51, J18, J43, J44, J45, J47, J84, J96, K29, K72, R93
	A2	13	77	Iron deficiency anaemia (D50)	D50, D64, I84, K21, K25, K27, K31, K52, K59, M10, N18, N19, N39
	A3	32	482	Cervical disc disorders (M50)	E11, E14, E72, E78, G20, G31, G45, G47, H25, H26, H81, I10, I11, I63, I65, I67, I69, I70, J32, J42, K76, K80, K82, M17, M47, M48, M50, M51, M81, M89, N28, N40
Female	B1	31	417	Disorders of glycoprotein metabolism (E77)	D50, D64, D69, D70, E03, E77, E79, I11, I27, I31, I38, I44, I47, I48, I49, I50, I51, J18, J42, J43, J44, J45, J47, J96, K27, K72, K80, M06, N18, N19, N28
	B2	36	576	Sleep disorders (G47)	E04, E11, E14, E78, F41, G31, G44, G45, G47, H25, H26, H81, I10, I63, I65, I67, I69, I70, J31, J32, K21, K25, K29, K31, K52, K59, K76, K82, M17, M47, M48, M50, M51, M81, M89, N39
35-59 years	C1	11	55	Disorders resulting from impaired kidney tubular function (N25)	B18, D64, D69, E77, I15, I31, K72, K74, N18, N19, N25
	C2	30	409	Menopausal and other perimenopausal disorders (N95)	E03, E04, E05, F41, F45, G44, G45, G47, H81, I67, I84, J31, J32, K21, K25, K29, K31, K52, K59, M06, M17, M47, M48, M50, M51, M81, M89, N39, N73, N95
	C3	18	148	Cerebral infarction (I63)	E11, E14, E78, E79, G31, I10, I11, I63, I65, I69, I70, K76, K80, K82, M10, N28, N40, R93
	C4	15	85	Other pulmonary heart diseases (I27)	I27, I42, I47, I48, I49, I50, I51, J18, J42, J43, J44, J45, J47, J96, Q24
60-69 years	D1	17	122	Respiratory failure, not elsewhere classified (J96)	C34, I27, J18, J42, J43, J44, J45, J47, J96, K21, K25, K29, K31, K82, N28, N40, R93
	D2	23	227	Unspecified kidney failure (N19)	D64, D69, E03, E11, E14, E77, E79, I11, I31, I38, I42, I44, I47, I48, I49, I50, I51, K72, K80, M06, M10, N18, N19
	D3	30	419	Sleep disorders (G47)	E04, E78, F41, G31, G44, G45, G47, H25, H26, H81, I10, I63, I65, I67, I69, I70, I84, J31, J32, K52, K59, K76, M17, M47, M48, M50, M51, M81, M89, N39
70-79 years	E1	37	584	Disorders of glycoprotein metabolism (E77)	C34, D50, D64, D69, E77, E79, G93, I27, I31, I38, I48, I50, I51, J18, J42, J43, J44, J45, J47, J84, J96, K21, K25, K27, K29, K31, K52, K72, K80, K82, M06, M10, N18, N19, N28, N40, R93
	E2	33	513	Other anxiety disorders (F41)	E03, E04, E11, E14, E72, E78, F41, G20, G31, G44, G45, G47, H25, H26, H81, I10, I11, I63, I65, I67, I69, I70, J32, K59, K76, M17, M47, M48, M50, M51, M81, M89, N39
	E3	4	6	Other conduction disorders (I45)	I44, I45, I47, I49
80+ years	F1	30	406	Hepatic failure, not elsewhere classified (K72)	D50, D64, D69, E46, E77, G93, I27, I31, I38, I48, I50, I51, J18, J43, J44, J45, J47, J84, J96, K21, K25, K27, K29, K31, K72, K80, K83, N19, N28, R93
	F2	34	545	Other nontoxic goitre (E04)	E03, E04, E11, E14, E72, E78, G20, G31, G45, G47, H25, H26, H81, I10, I11, I63, I65, I67, I69, I70, J32, J42, K52, K59, K76, M17, M47, M48, M50, M51, M81, M89, N39, N40
	F3	7	21	Other conduction disorders (I45)	E79, I44, I45, I47, I49, M10, N18

6 References

1. Chronic Condition Indicator (CCI) for ICD-10-CM (Beta Version) Available online: https://www.hcup-us.ahrq.gov/toolssoftware/chronic_icd10/chronic_icd10.jsp (accessed on 15 April 2021).
2. Westergaard, D.; Moseley, P.; Sørup, F.K.H.; Baldi, P.; Brunak, S. Population-Wide Analysis of Differences in Disease Progression Patterns in Men and Women. *Nat Commun* **2019**, *10*, 666, doi:10.1038/s41467-019-08475-9.

3. Menche, J.; Sharma, A.; Kitsak, M.; Ghiassian, S.D.; Vidal, M.; Loscalzo, J.; Barabási, A.-L. Disease Networks. Uncovering Disease-Disease Relationships through the Incomplete Interactome. *Science* **2015**, *347*, 1257601, doi:10.1126/science.1257601.
4. Hidalgo, C.A.; Blumm, N.; Barabási, A.-L.; Christakis, N.A. A Dynamic Network Approach for the Study of Human Phenotypes. *PLoS Comput Biol* **2009**, *5*, e1000353, doi:10.1371/journal.pcbi.1000353.
5. Park, J.; Lee, D.-S.; Christakis, N.A.; Barabási, A.-L. The Impact of Cellular Networks on Disease Comorbidity. *Mol Syst Biol* **2009**, *5*, 262, doi:10.1038/msb.2009.16.
6. Jeong, E.; Ko, K.; Oh, S.; Han, H.W. Network-Based Analysis of Diagnosis Progression Patterns Using Claims Data. *Sci Rep* **2017**, *7*, 15561, doi:10.1038/s41598-017-15647-4.
7. Katz, D.; Baptista, J.; Azen, S.P.; Pike, M.C. Obtaining Confidence Intervals for the Risk Ratio in Cohort Studies. *Biometrics* **1978**, *34*, 469, doi:10.2307/2530610.
